# Clinical Validity of Circulating Tumor DNA as Prognostic and Predictive Marker for Personalized Colorectal Cancer Patient Management

**DOI:** 10.3390/cancers14030851

**Published:** 2022-02-08

**Authors:** Ariane Hallermayr, Verena Steinke-Lange, Holger Vogelsang, Markus Rentsch, Maike de Wit, Christopher Haberl, Elke Holinski-Feder, Julia M. A. Pickl

**Affiliations:** 1 MGZ—Medizinisch Genetisches Zentrum, 80335 Munich, Germany; ariane.hallermayr@mgz-muenchen.de (A.H.); verena.steinke-lange@mgz-muenchen.de (V.S.-L.); elke.holinski-feder@mgz-muenchen.de (E.H.-F.); 2 Institute for Medical Information Processing, Biometry, and Epidemiology, Pettenkofer School of Public Health LMU Munich, 81377 Munich, Germany; 3 Medizinische Klinik und Poliklinik IV, Campus Innenstadt, Klinikum der Universität München, 80336 Munich, Germany; 4 Department of General, Visceral, Thoracic and Endocrine Surgery, Klinikum Garmisch-Partenkirchen, Teaching Hospital, Ludwig Maximilian University Munich, 82467 Garmisch-Partenkirchen, Germany; holger.vogelsang@klinikum-gap.de; 5 Department of General, Visceral and Thorax Surgery, Klinikum Ingolstadt, 85049 Ingolstadt, Germany; markus.rentsch@klinikum-ingolstadt.de; 6 Department of General, Visceral, Vascular and Transplant Surgery, University Hospital Munich, Campus Großhadern, Ludwig-Maximilians University of Munich, 81377 Munich, Germany; 7 Department of Haemaotology, Oncology and Palliative Medicine, Vivantes Klinikum Neukoelln, 12351 Berlin, Germany; maike.dewit@vivantes.de; 8 Department of Oncology, Vivantes Auguste-Viktoria-Klinikum, 12157 Berlin, Germany; 9 Department of Oncology and Hematology, Barmherzige Brüder, Klinikum St. Elisabeth, 94315 Straubing, Germany; christopher.haberl@klinikum-straubing.de

**Keywords:** ctDNA, cfDNA, residual disease, monitoring, colorectal cancer

## Abstract

**Simple Summary:**

CtDNA analysis is a promising tool in liquid biopsy for the detection of tumor recurrence and progression, and is increasingly adopted into clinical practice. Still, guidelines for the accurate clinical interpretation of ctDNA analysis results are largely lacking, especially for tumor mutant variants detected at very low frequencies. Here, we show that cutoff determination for the detection and quantification of low-frequency mutant variants enables the accurate prediction of residual disease, tumor recurrence and progression, even before clinical evidence. CtDNA analysis using these cutoffs outperformed cfDNA and CEA level measurements. With these findings, we highlight the need to thoroughly validate each liquid biopsy assay and define the assay-specific limit of blanks (LOB) and limit of quantifications (LOQ) of *BRAF* p.V600E and *KRAS* p.G12/p.G13 assays for clinical interpretation. Our approach enables accurate clinical interpretation to support clinical decision making.

**Abstract:**

Circulating tumor DNA (ctDNA) is a promising liquid biopsy (LB) marker to support clinical decisions in precision medicine. For implementation into routine clinical practice, clinicians need precise ctDNA level cutoffs for reporting residual disease and monitoring tumor burden changes during therapy. We clinically validated the limit of blank (LOB) and the limit of quantification (LOQ) of assays for the clinically most relevant somatic variants *BRAF* p.V600E and *KRAS* p.G12/p.G13 in colorectal cancer (CRC) in a study cohort encompassing a total of 212 plasma samples. We prove that residual disease detection using the LOB as a clinically verified cutoff for ctDNA positivity is in concordance with clinical evidence of metastasis or recurrence. We further show that tumor burden changes during chemotherapy and the course of disease are correctly predicted using the LOQ as a cutoff for quantitative ctDNA changes. The high potential of LB using ctDNA for accurately predicting the course of disease was proven by direct comparison to the routinely used carcinoembryonic antigen (CEA) as well as the circulating free DNA (cfDNA) concentration. Our results show that LB using validated ctDNA assays outperforms CEA and cfDNA for residual disease detection and the prediction of tumor burden changes.

## 1. Introduction

CfDNA is released from both tumor and normal cells into the circulation [1,2]. The presence and proportion of tumor-specific ctDNA in the entirety of cfDNA can be used as surrogates for tumor presence and overall tumor burden, and are analyzed through the measurement of tumor-specific mutant variants [3,4,5]. Besides ctDNA, other LB analytes including circulating tumor cells (CTCs) have also been investigated for clinical application. Whereas CTCs allow the extraction of detailed information at the single cell level [6,7], numerous studies have shown that ctDNA profiles are highly concordant with the molecular profile of primary tumors and metastases [8,9], and are considered to have a higher likelihood to enter clinical application [10]. Since CTCs and ctDNA are analytes present in LB, the advantages of LB, such as non-invasiveness and ease of repeatability, enable CTC and ctDNA analysis independently of the patient’s condition and at any desired time point [11,12,13]. In certain clinical courses of patients with non-small cell lung cancer (NSCLC) and breast cancer, LB using ctDNA analysis is already recommended to guide therapeutic decisions [14,15], and is even covered by health insurance. Furthermore, LB is expected to enter the clinical routine for CRC patient management in the near future, once clinical utility is proven for the following applications: (1) residual disease detection after surgery in CRC to facilitate decision on adjuvant therapy, (2) recurrence monitoring and (3) real-time monitoring of treatment response during chemotherapy.

Encouraging results of LB studies in these applications are highly desirable, as current standard procedures in CRC patient management, including CEA measurements, harbor several shortcomings. For example, the determination of prognosis after surgery based on CEA status combined with clinical-pathological characteristics of the resected primary specimen has limited predictive accuracy [16]. Furthermore, treatment monitoring and follow-up using CEA measurements, computed tomography (CT) scans and colonoscopies do not show an overall benefit [17], and harbor limited accuracy in recurrence prediction [18,19].

Before the implementation of ctDNA analysis into routine clinical practice, the diagnostic sensitivity and specificity of obtained variant-specific ctDNA measurement results must be clarified. CtDNA is often present at <1% variant allele frequency (VAF) in plasma, especially after surgery and chemotherapy initiation [3,20,21]. At these low levels of ctDNA, variant detection may be interfered with by the intrinsic noise of assays and by non-tumor specific signals due to clonal hematopoiesis. Hence, it may be difficult for clinicians to correctly interpret obtained ctDNA measurement results in the clinical context. Clinicians require variant-specific cutoffs for ctDNA positivity, which accurately predict residual disease and recurrence [3,22,23]. Furthermore, clinicians need precise quantification of ctDNA levels for correlation with tumor progression [23]. According to the “Protocols for Determination of Limits of Detection and Limits of Quantitation” (CLSI guidelines) [24], for the establishment of clinical laboratory measurement procedures, these detection and quantification cutoffs of clinical assays are obligatory in a clinical report, and are not equal to the general limit of detection (LOD) of the method, which is the only parameter commonly determined by LB assay providers.

To enable correct clinical interpretation of ctDNA analysis results, we determined the LOB and LOQ for each variant-specific ctDNA assay and aimed to validate these as cutoffs for ctDNA positivity and ctDNA quantification for usage in the detection of residual disease, recurrence and tumor burden monitoring by analyzing a total of 124 plasma samples of 22 CRC patients. We further compared the clinical validity of ctDNA analysis results with cfDNA and CEA concentration measurements.

## 2. Materials and Methods

### 2.1. Study Design and Participants

**Figure 1:**
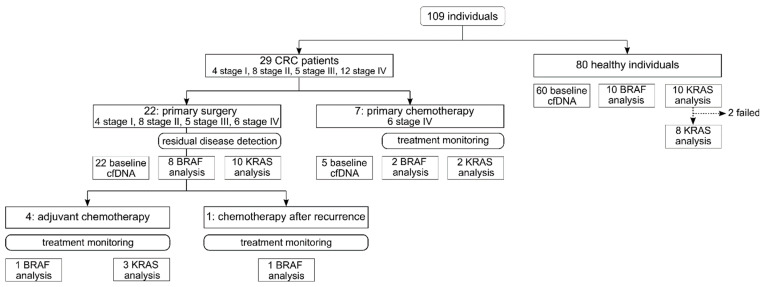
Study design. *BRAF* analysis indicates *BRAF* p.V600E analysis, and *KRAS* analysis represents analysis of one of the following variants: *KRAS* p.G12 [A/C/D/R/S/V] or *KRAS* p.G13D.

A total of 212 plasma samples were collected from 29 CRC UICC stage I-IV patients and 80 healthy individuals aged 19 to 87 years from October 2018 until March 2021 (Figure 1, Supplementary Tables S1 and S2) [25]. Patients prior to the initiation of therapy (surgery or chemotherapy) were included. The inclusion criteria for healthy individuals were no evidence for tumor predisposition, no previously diagnosed tumor, and no pregnancy. Since both *KRAS* and *BRAF* are localized on autosomes, the sex of healthy individuals was not considered as a co-morbidity. To account for age differences between the overall younger healthy controls and older patients, lymphocyte genomic DNA (gDNA) was analyzed to exclude clonal hematopoiesis in case of ctDNA positive status, which might be more frequent in elderly individuals [26].

Twenty-two out of 29 CRC patients underwent primary surgery. Baseline plasma samples were collected up to 5 days prior to primary surgery, and four to 50 days after surgery (Supplementary Data—Residual disease). Eighteen out of 22 had known tumor variant status (8 *BRAF* p.V600E and 10 *KRAS* p.G12/p.G13). In these 18 patients, respective ctDNA analysis was performed in plasma prior to and after surgery for residual disease detection (Supplementary Figures S1–S18). Four out of 18 patients underwent adjuvant chemotherapy (1 *BRAF* p.V600E and 3 *KRAS* p.G12/p.G13), and 1/18 patient received chemotherapy after disease recurrence. Treatment monitoring by ctDNA analysis was performed in all five cases.

Seven out of 29 CRC patients were treated with primary chemotherapy. Baseline plasma samples were collected up to five days prior to chemotherapy. Monitoring samples were collected at several time points throughout chemotherapy (Supplementary Figures S19–S27). Four out of seven had known tumor variant status (2 *BRAF* p.V600E and 2 *KRAS* p.G12/p.G13). Treatment monitoring using ctDNA analysis was conducted in all four cases.

ctDNA analysis was conducted in 18/29 patients (87 plasma samples) pre- and postoperatively using the LOB as distinct cutoff for residual disease detection. Further treatment was monitored in 9/29 patients (76 plasma samples) to assess whether the LOQ as a cutoff for ctDNA quantification can be used to reliably predict a response or resistance to chemotherapy.

Sample collection and preparation are described in the Supplementary Methods. This study was approved by the ethics commission of the Bavarian Medical Association (No. 17059) and was registered with the German registry for clinical trials (trial registration ID: DRKS00012890). Neither clinicians nor patients were informed about the results. All participants provided informed written consent prior to blood and tissue specimen collection.

### 2.2. Sample Preparation and Droplet Digital PCR

Information on DNA extraction and ddPCR protocols is provided in the Supplementary Methods.

### 2.3. Droplet Digital™ PCR

Droplet Digital PCR (ddPCR) was performed using the single Probe ddPCR *BRAF* p.V600E assay (Bio-Rad, Hercules, CA, USA, #dHsaMDV2010027) and the *KRAS* p.G12/p.G13 screening kit (Bio-Rad, #1863506) on the QX200 system (Bio-Rad) according to the manufacturer’s instructions (see also Supplementary Methods). The *KRAS* p.G12/p.G13 screening kit (Bio-Rad) provides a positive signal if one of seven variants is present, but cannot specify which of the seven analyzed variants is actually present. To obtain reliable results, 20 to 30 ng of cfDNA was analyzed per reaction. Gating was performed based on mutant variant and wild-type (WT) control samples, and variant populations were identified using the QX Manager software (Bio-Rad, v.1.1). ctDNA was quantified in terms of the mutant VAF, which describes the abundance of detected mutant alleles within all detected alleles and is calculated as follows:

(1) $$\mathrm{VAF}\;\left(\%\right)=\frac{N_{\mathrm{mut}}}{N_{\mathrm{mut}}+N_{\mathrm{WT}}}\times 100$$

Equation (1). Variant allele frequency. VAF: variant allele frequency; N_mut_: number of mutant alleles; N_WT_: number of wild type alleles.

Samples with VAFs > LOB were defined with ctDNA positive status, and samples with VAFs > LOQ harbored quantifiable ctDNA VAFs.

### 2.4. Determination of Cutoffs for ctDNA Positive Status and Quantifiable ctDNA

The LOB and LOQ of ddPCR assays were determined with 95% confidence intervals in accordance with CLSI guidelines [24]. As the determination of the LOB is based on the detection of false positive results in negative controls, the 95% confidence interval at this threshold describes 95% specificity of the assays. Accordingly, ≥60 WT controls were measured with each assay to determine the LOB at 0.02 and 0.11% VAF for *BRAF* pV600E and *KRAS* p.G12/p.G13 assays, respectively. Furthermore, ≥40 replicates of positive controls containing the targeted variant with a VAF of the tentative LOQ were measured to determine the LOQ with achieving at least 80% precision and 90% trueness, at 0.52 and 0.41% VAF for *BRAF* pV600E and *KRAS* p.G12/p.G13 assays, respectively. Cutoffs were validated for 20 to 30 ng of input DNA [27].

### 2.5. Determination of Cutoff for Elevated cfDNA Concentrations

Previous studies described elevated plasma cfDNA concentrations in CRC patients [28,29,30,31]. To test whether plasma cfDNA concentration can add useful information to the biomarker portfolio in CRC, plasma cfDNA concentrations of 60 healthy individuals were compared to those in 128 samples from 29 CRC patients, which were quantified using the High-Sensitivity NGS Fragment Analysis Kit (Agilent, Santa Clara, CA, USA, #DNF-474-0500) on the Fragment Analyzer system (Agilent) (Supplementary Tables S2 and S3). According to CLSI guidelines [24], a minimum sample size of 60 is required for establishing the LOB with a 95% confidence interval. Therefore, including 60 healthy individuals for establishing the LOB as a cutoff for elevated cfDNA concentration is considered robust. With a median plasma cfDNA concentration of 2.5 ng/mL in healthy individuals and 11.6 ng/mL in CRC patients at baseline, significantly higher cfDNA concentrations were observed in CRC patients (*p*-value: 2.64 × 10^−11^, Wilcoxon test) (Supplementary Figure S28). With 95% specificity, a cutoff at 5.6 ng/mL cfDNA was established to differentiate between CRC patients and healthy individuals, as follows:

(2) $$\mathrm{LOB}=\mathrm{Result}\;\mathrm{at}\;\mathrm{position}\left[0.95\times N_{B}+0.5\right]=P_{1-\alpha }$$

Equation (2). Determination of the cfDNA cutoff. LOB: limit of blank; N_B_: number of negative control measurements; P_(1−α)_: percentile at the level of 1 − α.

Linear interpolation between the results of the next lower and the next higher rank position was used to determine the cfDNA cutoff [24].

### 2.6. CEA Analysis

CEA levels in plasma were determined using the Human CEA ELISA Kit (Biorbyt, Cambridge, UK, Cat# orb438561) in accordance with the manufacturers’ instructions.

### 2.7. Statistical Analysis

Differences between the cfDNA concentration of healthy individuals and CRC patients were determined using a Wilcoxon test. Differences in ctDNA VAF, cfDNA and CEA concentrations depending on time in the course of the disease were calculated using a Kruskal–Wallis test. Bonferroni correction was used to adjust p-values for multiple testing. Using a priori power and sample size analysis, the minimum required sample sizes for a power of 0.8 were determined to be 18 and 9 per group for the Wilcoxon test and the Kruskal–Wallis test, respectively (G*Power version 3.1.94, https://gpower.software.informer.com/3.1/, accessed on 3 January 2022). *p*-values < 0.05 were considered statistically significant. All statistical analyses were performed using the stats R package in R version 4.0.3 (https://www.r-project.org/, accessed on 3 January 2022).

## 3. Results

### 3.1. Cutoff Validation for ctDNA Status and Quantifiable ctDNA VAFs in Reference Materials

**Figure 2:**
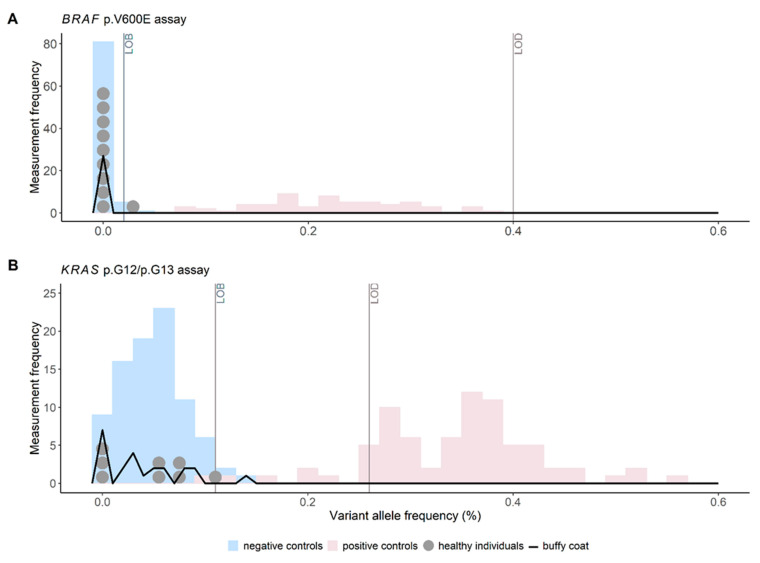
Cutoff for positive ctDNA status (i.e., LOB) is clinically verified by ctDNA measurements of healthy control samples (grey dots), as ctDNA signals of healthy controls are generally below the set ctDNA positivity cutoff of (**A**) *BRAF* p.V600E and (**B**) *KRAS* p.G12/p.G13 assays. Clonal hematopoiesis does not interfere with true tumor-derived positive ctDNA status, as ctDNA signals from buffy coat-containing lymphocyte DNA are generally below LOB (black line). Histogram of measurement results of negative (blue) and positive control (pink) reference material measurements allow definition of the LOB and LOD, respectively, as described in the analytical validation [27].

Precise cutoff definition of positive ctDNA status in a plasma sample is a prerequisite for the ctDNA-based detection of residual disease and recurrence, and was determined previously using well-characterized WT control reference materials according to CLSI guidelines [24]. In our study, samples are defined as ctDNA-positive when ctDNA VAFs of *BRAF* p.V600E or *KRAS* p.G12/p.G13 variants are detected above the respective LOB (at 0.02 and 0.11% VAF, respectively, Figure 2 in blue) [27]. In the case of *KRAS* p.G12/p.G13 variants, the assay cannot differentiate between the targeted variants, and therefore a singular cutoff was validated.

Exact definition of the cutoff, from which ctDNA VAFs can be quantified, is required for monitoring quantitative tumor changes during treatment, and was defined previously using positive control reference materials according to CLSI guidelines [24,27]. This cutoff is defined as LOQ. In this study, quantitative ctDNA VAFs can be assessed from samples with *BRAF* p.V600E and *KRAS* p.G12/p.G13 ctDNA VAFs above LOQ (at 0.52 and 0.41% VAF, respectively).

### 3.2. Cutoff Verification for ctDNA Status in Plasma of Healthy Controls

To clinically verify the variant-specific ctDNA positivity cutoff, previously validated using reference materials, ten healthy control samples were analyzed with *KRAS* p.G12/p.G13 and *BRAF* p.V600E assays, respectively (Figure 2).

Notably, as two *KRAS* p.G12/p.G13 analyses failed, the results of the measurement of eight *KRAS* p.G12/p.G13 considered. None (zero out of eight) and 10% (1/10) of the healthy control samples exceeded the previously validated ctDNA positivity cutoffs for the *KRAS* p.G12/p.G13 and *BRAF* p.V600E assays, respectively. In detail, the single measured VAF of the healthy control sample exceeding the cutoff of the *BRAF* p.V600E assay was 0.03%. By definition, a maximum of 15% of the healthy control samples may exceed the cutoff for verification [24]. Since clinical cutoff verification was conducted in the same setting as analytical validation and the criteria for cutoff verification are met, the ctDNA positivity cutoff was clinically verified for both *KRAS* p.G12/p.G13 and *BRAF* p.V600E assays (Figure 2, grey dots). Consequently, cutoffs could be used for residual disease and recurrence analysis.

### 3.3. Positive ctDNA Status Is Tumor Specific

The number of non-tumor-derived positive ctDNA signals in plasma samples of CRC patients must be thoroughly determined. To that the detected ctDNA signals in the plasma of CRC patients are non-tumor-specific and actually a result of clonal hematopoiesis [32], *BRAF* p.V600E and *KRAS* p.G12/p.G13 analyses were performed on lymphocyte gDNA of all CRC patients with ctDNA-positive plasma samples (Figure 2, black line). Zero out of 27 *BRAF* and only 1/23 *KRAS* signals were detected above the cutoff for ctDNA positivity (i.e., >LOB) in these samples. As in this single case of a positive lymphocyte gDNA signal, the VAF was lower than in the plasma sample (0.14 vs. 1.48% (±0.39%)), the respective plasma was still considered as ctDNA-positive. Overall, these results indicate that clonal hematopoiesis did not perturbate ctDNA results, and that positive plasma ctDNA status was indeed tumor specific.

### 3.4. At Baseline, Elevated cfDNA Concentration Outperform ctDNA Positive Status and CEA Levels

**Figure 3:**
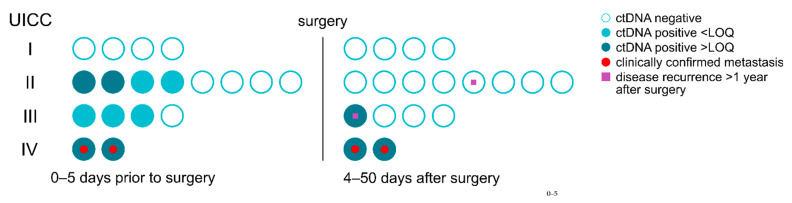
Determination of ctDNA positivity status in CRC stage I–IV patients for residual disease detection.

Using the ctDNA positivity cutoffs, positive ctDNA status was observed in 9/18 patients at baseline. Positive ctDNA status rate increased from early to late UICC stage (i.e., zero out of four stage I, four out of eight stage II, three out of four stage III, and two out of two stage IV) (Figure 3, Supplementary Methods—Residual disease). These observations are in line with previous studies showing that higher tumor stages are expected to release more ctDNA into circulation [22,28,29,30].

As a reference biomarker to ctDNA, the commonly used CRC marker CEA was measured in the plasma of CRC patients. Elevated CEA levels (in CRC patients compared to healthy individuals) were defined according to the literature as values above 2.5 and 5 ng/mL in non-smokers and smokers, respectively [33,34]. At baseline, CEA levels were elevated in only 2/18 patients (i.e., zero out of four stage I, zero out of eight stage II, one out of four stage III, and one out of two stage IV).

For the analysis of total cfDNA, a clinical cutoff of 5.6 ng/mL cfDNA in plasma was established for elevated cfDNA concentration (in CRC patients compared to healthy individuals) (Methods, Supplementary Figure S1). At baseline, elevated cfDNA concentrations were observed in 12/18 patients (i.e., two out of four stage I, four out of eight stage II, four out of four stage III, and two out of two stage IV). Since both ctDNA and elevated plasma cfDNA concentration are detected with 95% specificity, results can be compared and indicate that cfDNA is a better singular diagnostic marker at baseline than ctDNA and CEA. The combination of the three markers did not improve the detection rate of CRC at baseline.

### 3.5. Residual Disease and Recurrence Are Predicted by ctDNA Positive Status

To test the association of ctDNA-positive status in plasma with residual disease detection after surgery, VAFs of *BRAF* p.V600E and *KRAS* p.G12/p.G13 variants were measured in cfDNA from plasma of 18 CRC patients prior to surgery (i.e., baseline sample) and four to 50 days after surgery (Figure 3). All 18 patients underwent locoregional R0 resection.

**Figure 4:**
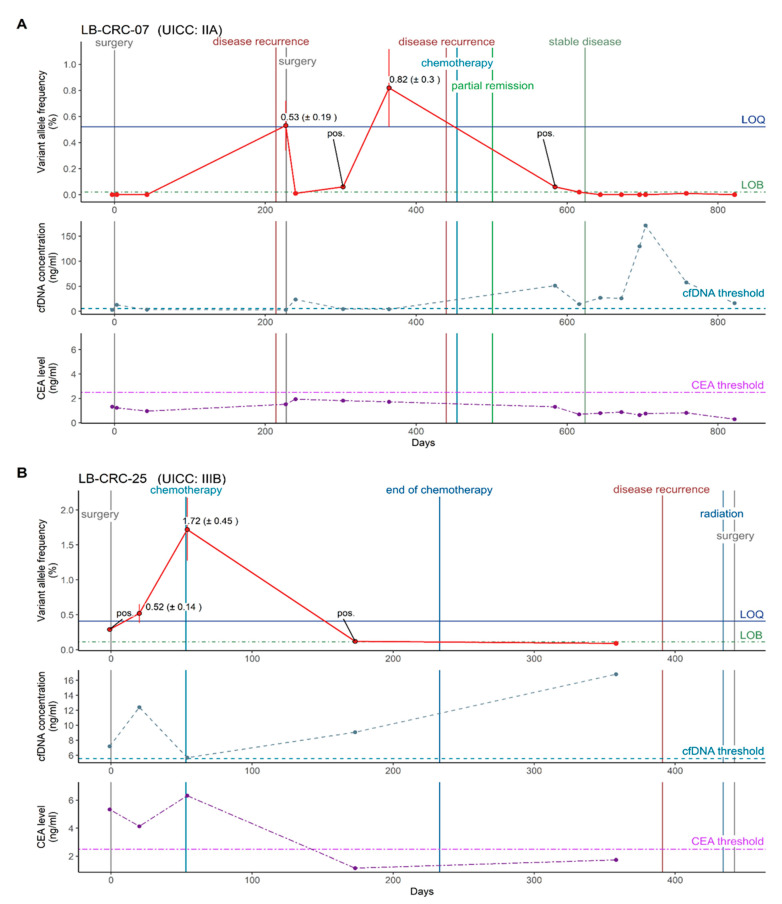
ctDNA (line 1), cfDNA (line 2) and CEA (line 3) analysis throughout the course of the disease in two CRC patients (**A**) LB-CRC-07 and (**B**) LB-CRC-25, with recurrence.

At baseline, the mutant variants were detected in half of the CRC patients (i.e., VAF > LOB, 9/18): one out of fourstage II, one out of one stage III and one out of one stage IV patients with *BRAF* and three out of four stage II, two out of three stage III and one out of one stage IV patients with *KRAS* variants. As expected, the nine patients with negative ctDNA status prior to surgery were also negative after surgery. These CRC patients with negative ctDNA status did not have metastasis from baseline up to 50 days after surgery, indicating high diagnostic specificity of ctDNA results. Notably, one of these patients later had clinically recurrent disease twice, which could be predicted by ctDNA detection up to three months prior to clinical evidence (Figure 3 and Figure 4A, LB-CRC-07).

Of the nine patients with positive ctDNA status at baseline, three remained ctDNA-positive after surgery (one out of three stage III, and two out of two stage IV patients; all > LOQ), i.e., molecular residual disease (MRD), defined by the presence of ctDNA in plasma, could be identified. The two stage IV patients with MRD had clinically confirmed metastasis. The stage III patient with MRD had no confirmed metastasis, but was classified as a high-risk patient and therefore received adjuvant chemotherapy, which resulted in the disappearance of ctDNA during the course of treatment. This patient had recurrence more than one year after surgery and ~six months after the completion of adjuvant chemotherapy (Figure 3 and Figure 4B, LB-CRC-25). Although disease recurrence could not be predicted around one month prior to clinical evidence in one patient, overall, these results indicate the potential of ctDNA analysis as a complementary marker for MRD and recurrence detection.

### 3.6. Residual Disease and Recurrence Are More Reliably Predicted by Positive ctDNA Status Than by Elevated cfDNA Concentration and CEA Levels

To estimate the predictive accuracy of positive ctDNA status for residual disease and recurrence detection, results were compared to cfDNA and CEA. MRD was detected in 3/18 patients, and recurrence occurred in 2/18 patients treated with primary surgery. Two of the three patients with MRD had clinically confirmed metastasis. In one of these two patients, the cfDNA concentration was elevated, whereas CEA levels were within the normal range from baseline until one month after surgery. Accordingly, besides ctDNA, cfDNA but not CEA predicted MRD in this patient (LB-CRC-38, Supplementary Figure S17). In the second patient, cfDNA concentration could not be assessed in the period of four to seven weeks after surgery. CEA levels were elevated at baseline and two weeks after surgery, indicating MRD (LB-CRC-09, Supplementary Figure S6). In the third patient with MRD, recurrence occurred more than one year after surgery and ~six months after completion of adjuvant chemotherapy. Plasma cfDNA concentration was elevated throughout the entire course of the disease. As no qualitative changes of the cfDNA marker were observed over time, neither MRD nor recurrence could be predicted by cfDNA measurement in this patient. CEA levels were elevated in this patient from baseline until two months after surgery, but were in the normal range one and seven months before recurrence. Therefore, CEA predicted MRD but not disease recurrence in this patient (LB-CRC-25, Figure 4B), analogous to ctDNA. In the second patient with recurrence, cfDNA concentration was within the normal range from baseline throughout the first year of follow-up. With the initiation of chemotherapy for the treatment of systemic nodal progression, cfDNA concentration was elevated, but not before or in parallel (zero days to 2.5 months) to clinically evident recurrence. CEA levels were within the normal range from baseline throughout the course of disease. Hence, in contrast to ctDNA, both biomarkers did not predict disease recurrence (LB-CRC-07, Figure 4A). Taken together, cfDNA predicted residual disease in one of the patients with MRD and CEA in two of them, but could not predict recurrence in any case.

These results indicate higher sensitivity of ctDNA compared to cfDNA and CEA for detection of MRD and recurrence.

### 3.7. Chemotherapy Monitoring Possible through Precise ctDNA Quantification

In contrast to residual disease and recurrence detection, for which ctDNA-positive status in plasma is determined, quantitative changes in ctDNA VAF are analyzed for chemotherapy monitoring.

Treatment was monitored in nine CRC patients to assess whether quantitative changes in ctDNA VAF can predict response or resistance to chemotherapy.

Patients with primary chemotherapy: Four out of nine patients who received primary chemotherapy (LB-CRC-02, LB-CRC-30, LB-CRC-32, LB-CRC-43) were diagnosed with stage IV CRC (two out of four *BRAF* and two out of four *KRAS* variants). At baseline, ctDNA VAFs of these four patients ranged from 8.99 (±2.36%) to 47.75% (±7.11%), and significantly decreased within the first month of treatment (undetectable to 12.19% (±1.82%)), indicating a good response to chemotherapy. In all of these patients, decreases or increases in ctDNA VAF during the course of treatment were associated with a response or resistance to chemotherapy.

Patients with adjuvant chemotherapy: Four out of nine patients were treated with adjuvant chemotherapy following primary surgery (three out of four stage III with *KRAS* and one out of four stage IV with *BRAF* variants). MRD was detected in only two of those four patients after surgery and before the initiation of adjuvant chemotherapy. The decrease in ctDNA VAF from 1.72 (±0.45%) and 2.01% (±0.73%), respectively, to undetectable ctDNA was associated with a good response to treatment in both patients (LB-CRC-38, LB-CRC-25). In the remaining two patients, no MRD was detected after surgery and before initiation of adjuvant chemotherapy. For these patients, no positive ctDNA signals were detected in follow-up samples collected within one year (LB-CRC-18, LB-CRC-29).

Patient with palliative chemotherapy: In one out of nine stage II patients (*BRAF* variant) who received chemotherapy after diagnosis of systemic nodal progression more than one year after primary surgery (LB-CRC-07), ctDNA VAF decreased from 0.82% (±0.30%) to undetectable ctDNA alongside partial remission and stable disease.

Overall, the precise quantification of ctDNA VAF enabled the association of increasing ctDNA VAF to resistance and decreasing ctDNA VAF to response to chemotherapy in all patients.

### 3.8. Chemotherapy Monitoring by Precise ctDNA Quantification Outperforms cfDNA Concentration and CEA Levels

To evaluate the predictive accuracy of quantitative changes in ctDNA VAFs for predicting response or resistance to chemotherapy, results were compared to cfDNA and CEA. Before the initiation of chemotherapy, ctDNA was detected in seven out of nine patients with ctDNA VAFs ranging from 0.52 (±0.14%) to 47.75% (±7.11%). In all of these patients, decreases or increases in ctDNA VAFs during the course of treatment were associated with a response or resistance to chemotherapy.

Patients without quantifiable ctDNA before chemotherapy: The two patients without detectable ctDNA before and during chemotherapy (stage III, *KRAS* variants) showed increased cfDNA levels before the initiation of chemotherapy. While in one of these two patients cfDNA levels decreased to the normal range within one year (LB-CRC-18), the cfDNA levels in the second patient oscillated during chemotherapy and were elevated again one year after treatment initiation (LB-CRC-29). In both patients, CEA levels were within the normal range throughout chemotherapy. Therefore, cfDNA and CEA could not support prediction of response or resistance to chemotherapy in both patients (Supplementary Figures S22 and S24).

Patients with quantifiable ctDNA before chemotherapy: In one patient (stage II at diagnosis, *BRAF* variant), cfDNA concentration was elevated only after the initiation of chemotherapy, which was inconsistent with the clinical findings of partial remission and stable disease. CEA levels were within the normal range throughout the course of chemotherapy (LB-CRC-07, Figure 4A). One patient (stage III, *KRAS* variant) showed an elevated cfDNA concentration throughout the course of chemotherapy. CEA levels were elevated before initiation and decreased to the normal range during the course of chemotherapy (LB-CRC-25, Figure 4B). The remaining five out of seven patients were diagnosed with stage IV CRC (three out of five *BRAF* variants, two out of five *KRAS* variants). In one of these patients, cfDNA concentration and CEA levels could only be assessed in samples collected following progressive disease. Both markers were elevated in all of these samples and correlated well with the clinical finding of progressive disease (LB-CRC-02, Supplementary Figure S20). In another stage IV patient, the cfDNA concentration was elevated before initiation, decreased to the normal range after two months of chemotherapy, and was elevated again approximately two months later, correlating well with clinical findings of stable and progressive disease. CEA levels were elevated throughout the entire course of disease (LB-CRC-30, Supplementary Figure S25). In the third stage IV patient, the cfDNA concentration was elevated in all but one sample throughout the course of chemotherapy. CEA levels were elevated before the initiation of chemotherapy and decreased to the normal range approximately 1.5 months after ctDNA became undetectable for the first time (LB-CRC-32, Supplementary Figure S26). In the remaining two stage IV patients, cfDNA concentration was elevated throughout the course of chemotherapy. In one of the two patients, CEA levels were within the normal range in all but one sample that did not harbor ctDNA, whereas in the second patient, CEA levels were within the normal range throughout chemotherapy (LB-CRC-38 and LB-CRC-43, Supplementary Figures S27 and S28). Taken together, cfDNA predicted response or resistance to chemotherapy in only two and CEA in only three of the patients for whom ctDNA VAFs could be correlated with response or resistance to chemotherapy.

These results indicate the higher sensitivity of ctDNA quantification compared to cfDNA and CEA for predicting response or resistance to chemotherapy and therefore suggest that ctDNA is a more suitable marker than cfDNA and CEA.

### 3.9. Significant Differences Depending on Time in Course of Disease in ctDNA VAFs, but Not in cfDNA Concentration and CEA Levels

**Figure 5:**
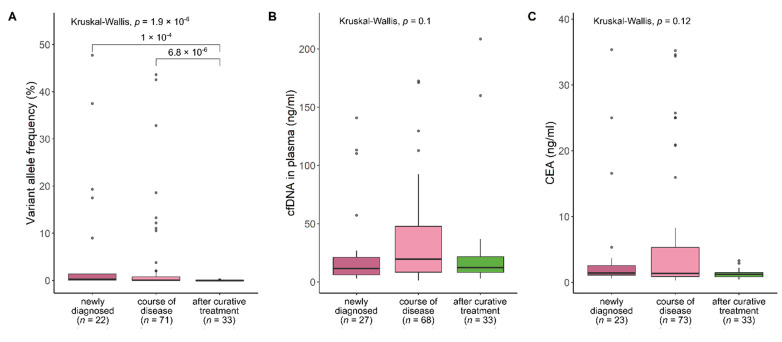
ctDNA VAFs, cfDNA concentration and CEA levels at different times in the course of the disease. (**A**) ctDNA quantification levels but not (**B**) cfDNA concentration and (**C**) CEA levels significantly differ at different times in the course of disease.

To test whether the observed differences in ctDNA VAFs, cfDNA concentration and CEA levels depending on the course of disease are statistically significant, all three markers were compared between samples collected at baseline, during the course of disease with clinically evident tumor, and during follow-up after curative treatment. Indeed, significant differences in ctDNA VAFs were identified depending on sampling time (baseline, course of disease, after curative treatment) (*p* = 1.9 × 10^−6^, Figure 5A). In contrast, no significant differences were observed in cfDNA concentration or CEA levels (*p* = 0.1 and 0.12, respectively, Figure 5B,C). These results strongly suggest that accurate detection of positive ctDNA status and precise quantification of ctDNA VAFs using well-defined mutant-specific LOBs and LOQs reflect the generally increased tumor burden at baseline and during the course of disease, as well as the absence of tumors in follow-up samples after curative treatment (*p* = 1 × 10^−4^ and 6.8 × 10^−6^, respectively). In contrast, cfDNA concentration and CEA levels do not an detect increased tumor burden or the absence of tumors with comparable sensitivity. These data are consistent with results for residual disease and recurrence detection and for chemotherapy monitoring.

## 4. Discussion

CtDNA analysis enables the non-invasive and real-time assessment of tumor burden and mutant signatures in cancer patients, which may be used for residual disease, recurrence and tumor progression detection. For implementation into clinical routine, clinically relevant positive ctDNA signals at low levels must clearly be verified as being tumor-specific without interference from analytical measurement noise (technical artefact) or clonal hematopoiesis (biological artefact) [35]. Hence, clinicians need a defined cutoff for true tumor-specific ctDNA positivity. In this study, we defined and clinically verified a cutoff for ctDNA positivity according to the CLSI guidelines [24,27], and showed that only tumor samples but not healthy controls exceeded this cutoff. We further showed that clonal hematopoiesis did not interfere with positive ctDNA results in plasma samples. These results demonstrate that ctDNA analysis should be thoroughly validated, as with other clinical test procedures, and that obtained ctDNA signals above the defined ctDNA positive cutoff are indeed tumor-specific. This is an advantage over non-tumor-specific CEA and cfDNA markers in the plasma of CRC patients, which are generally present in healthy individuals.

We clinically validated the positive ctDNA status for the assessment of residual disease and recurrence. To validate LB for residual disease, we analyzed 18 CRC patients up to 50 days after primary surgery. We detected MRD in three of these patients, two of them having metastatic disease and one of them without clinical evidence of recurrence at the time of measurement. We found that positive ctDNA status is a better prognostic marker than the alternative liquid biopsy marker cfDNA and the commonly used CRC marker CEA. These results are in line with data from a pooled analysis of cohort studies showing that the prognostic accuracy of CEA and other commonly used markers is only ~50–60% [16]. In contrast, residual disease detection with postsurgery ctDNA status outperformed all routinely used markers with a prognostic accuracy of ~70%. The combination of ctDNA status with all standard clinical-pathological markers resulted in ~80% prognostic accuracy [16].

To validate LB for recurrence, we monitored patients throughout the course of disease and correlated signals above the ctDNA positivity cutoff with clinically evident recurrence. Our data suggest that ctDNA analysis may be meaningful in surveillance during follow-up, particularly as it outperformed the routinely used CEA marker. Furthermore, recurrence was detected by ctDNA up to three months before clinical evidence. These findings are consistent with the results published by Reinert et al. and Wang et al. [36,37]. CtDNA analysis to support current approaches may be valuable, as a large meta-analysis including 19 studies showed that current follow up strategies including CEA, CT scans and colonoscopies do not improve overall survival, CRC-specific survival or relapse-free survival. Conversely, more intensive follow-ups are likely to increase harms and costs [38]. In contrast, non-invasive liquid biopsies are likely to add accuracy to follow-up measurements, thus enabling the detection of recurrence several months before standard measurements [16].

Besides the qualitative assessment of ctDNA positivity, it is necessary to define a ctDNA quantification cutoff at a low level, from which reliable and precise determination of ctDNA VAFs is possible. Precise quantification with 95% confidence interval from LOQ to 100% ctDNA VAF is a prerequisite for the reliable interpretation of decreases or increases [27]. The LOQ enables clinicians to identify true VAF changes, a prerequisite for tumor monitoring during chemotherapy. We used our previously defined LOQ as a cutoff for ctDNA VAF quantifiability, and show that changes in VAFs above LOQ actually reflect tumor progression and response to therapy in all cases, thereby outperforming cfDNA and CEA measurements. These data are in line with previous data investigating standard approaches including CEA for treatment monitoring. Specifically, a meta-analysis comprising 52 studies concluded that CEA should be supplemented by another method due to poor performance [39]. Our data, in line with others [36,37,40,41], suggest that ctDNA can add valuable information on tumor progression and the response to treatment.

Although cutoffs for ctDNA-positive status and ctDNA quantifiability are assay-specific, our results indicate that defining cutoffs is critical for sensitive and specific clinical interpretation of ctDNA analysis results. Cutoffs for other assays can be easily validated according to the protocol described here. Validation of specific cutoffs for ctDNA-positive status and ctDNA quantification is an essential step towards implementation into clinical practice.

Our data show that elevated cfDNA concentration may be a valuable supporting marker for diagnosis of CRC, as cfDNA levels were elevated in 12/18 CRC patients at baseline. Thereby, the detection rate was slightly higher when compared to positive ctDNA status (9/18 detected) and clearly more sensitive than CEA (2/18 detected). Taken together, both liquid biopsy markers, cfDNA concentration and ctDNA status and VAFs, may add value to clinical practice, with cfDNA concentration being a supportive diagnostic marker and ctDNA status and VAFs being informative of residual disease, recurrence and tumor progression.

Nevertheless, our study has some limitations that should be considered. First, the small sample size of CRC patients included represents a limitation. However, by analyzing a large number of plasma samples over a long period of time and by applying extensively validated cutoffs for residual disease detection and treatment monitoring, we were able to thoroughly characterize all of our patients by obtaining strong evidence regarding the potential of ctDNA analysis for disease monitoring. This strong evidence can be further supported by the concordance of our results with previously published data. Future studies on recurrence and treatment monitoring may use our approach of accurately defined cutoffs in a larger cohort.

Second, the inclusion of patients at all stages could reduce the power to identify relevant differences between these disease stages in CRC patients. However, our main goal is to demonstrate the importance of distinct cutoffs for the clinical interpretation of ctDNA analysis, regardless of disease stage. Therefore, the inclusion of all stages does not impact our findings.

Third, analyzing only the *BRAF* and *KRAS* hotspot variants limits the benefits of this method to patients carrying one of these variants in tumor tissue. Still, since approximately 10 and 34% of CRC cases harbor the *BRAF* and *KRAS* hotspot variants, respectively [42], a large proportion of CRC patients will benefit from this highly sensitive and cost-effective ctDNA analysis method. Moreover, testing for hotspot variants in these two genes is recommended for metastatic CRC patients, which may facilitate the clinical application of *BRAF* and *KRAS* hotspot variants in LB [43]. To extend the benefits of ctDNA analysis to all CRC patients, validated untargeted ctDNA analysis would be required in the future. Furthermore, combining ctDNA with additional analytes present in LB might improve the patient management of more CRC patients. CTCs may represent a useful predictive and prognostic marker [44,45,46,47]. Recent advances in CTC-based technologies even enable genome-wide analysis of CTCs [6,7,48]. Furthermore, the differential expression of exosomal miRNAs such as miRNA-21 or miRNA-345 could be identified as potential prognostic markers in CRC [46,47,49,50,51]. Specific miRNAs such as miRNA-21 could even be identified as potential new targets for the treatment of drug resistance [46,47,52].

Fourth, the age difference of our control cohort and CRC patients might introduce potential bias due false positive signals in the older CRC cohort due to age-related clonal hematopoiesis. Therefore, we analyzed lymphocyte gDNA to exclude false positive signals originating from clonal hematopoiesis [26].

## 5. Conclusions

In conclusion, our LOB- and LOQ-based approach for ctDNA detection and quantification in CRC patients is an accurate approach and is easy to implement in clinical care for the prediction of residual disease, disease recurrence and treatment monitoring as a supplement to current approaches such as CEA.

## Data Availability

The data presented in this study are available within the article and its supplementary data files.

## Supplementary Materials

The following supporting information can be downloaded at: https://www.mdpi.com/article/10.3390/cancers14030851/s1, Supplement: Supplementary Methods and Data; Table S1: Baseline characteristics of study participants, Table S2: Data of all samples collected from study participants, Table S3: Data used for the determination of the cutoff for elevated cfDNA concentrations, Figure S1: Residual disease detection in patient LB-CRC-03, Figure S2: Residual disease detection in patient LB-CRC-05, Figure S3: Residual disease detection in patient LB-CRC-06, Figure S4: Residual disease detection in patient LB-CRC-07, Figure S5: Residual disease detection in patient LB-CRC-09, Figure S6: Residual disease detection in patient LB-CRC-10, Figure S7: Residual disease detection in patient LB-CRC-11, Figure S8: Residual disease detection in patient LB-CRC-14, Figure S9: Residual disease detection in patient LB-CRC-18, Figure S10: Residual disease detection in patient LB-CRC-19, Figure S11: Residual disease detection in patient LB-CRC-23, Figure S12: Residual disease detection in patient LB-CRC-24, Figure S13: Residual disease detection in patient LB-CRC-25, Figure S14: Residual disease detection in patient LB-CRC-29, Figure S15: Residual disease detection in patient LB-CRC-37, Figure S16: Residual disease detection in patient LB-CRC-38, Figure S17: Residual disease detection in patient LB-CRC-40, Figure S18 Residual disease detection in patient LB-CRC-41, Figure S19: Monitoring of mutant variant, CEA levels and cfDNA concentration in patient LB-CRC-02, Figure S20: Monitoring of mutant variant, CEA levels and cfDNA concentration in patient LB-CRC-07, Figure S21: Monitoring of mutant variant, CEA levels and cfDNA concentration in patient LB-CRC-18, Figure S22: Monitoring of mutant variant, CEA levels and cfDNA concentration in patient LB-CRC-25, Figure S23: Monitoring of mutant variant, CEA levels and cfDNA concentration in patient LB-CRC-29, Figure S24: Monitoring of mutant variant, CEA levels and cfDNA concentration in patient LB-CRC-30, Figure S25: Monitoring of mutant variant, CEA levels and cfDNA concentration in patient LB-CRC-32, Figure S26: Monitoring of mutant variant, CEA levels and cfDNA concentration in patient LB-CRC-38, Figure S27: Monitoring of mutant variant, CEA levels and cfDNA concentration in patient LB-CRC-43, Figure S28: Measurements of plasma cfDNA concentration in healthy individuals and newly diagnosed CRC patients for establishment of a CRC-specific cutoff for cfDNA elevation.

## References

[1] Lui Y.Y.N., Chik K.-W., Chiu R.W.K., Ho C.-Y., Lam C.W.K., Lo Y.D. (2002). Predominant Hematopoietic Origin of Cell-free DNA in Plasma and Serum after Sex-mismatched Bone Marrow Transplantation. Clin. Chem..

[2] Chen E., Cario C.L., Leong L., Lopez K., Márquez C.P., Chu C., Li P.S., Oropeza E., Tenggara I., Cowan J. (2021). Cell-free DNA concentration and fragment size as a biomarker for prostate cancer. Sci. Rep..

[3] Diehl F., Schmidt K., Choti M.A., Romans K., Goodman S., Li M., Thornton K., Agrawal N., Sokoll L., Szabo S.A. (2008). Circulating mutant DNA to assess tumor dynamics. Nat. Med..

[4] Momtaz P., Gaskell A.A., Merghoub T., Viale A., Chapman P.B. (2014). Correlation of tumor-derived circulating cell free DNA (cfDNA) measured by digital PCR (DigPCR) with tumor burden measured radiographically in patients (pts) with BRAFV600E mutated melanoma (mel) treated with RAF inhibitor (RAFi) and/or ipilimumab (Ipi). J. Clin. Oncol..

[5] Schøler L.V., Reinert T., Ørntoft M.-B.W., Kassentoft C.G., Árnadóttir S.S., Vang S., Nordentoft I., Knudsen M., Lamy P., Andreasen D. (2017). Clinical Implications of Monitoring Circulating Tumor DNA in Patients with Colorectal Cancer. Clin. Cancer Res..

[6] Carter L., Rothwell D.G., Mesquita B., Smowton C., Leong H.S., Fernandez-Gutierrez F., Li Y., Burt D.J., Antonello J., Morrow C.J. (2017). Molecular analysis of circulating tumor cells identifies distinct copy-number profiles in patients with chemosensitive and chemorefractory small-cell lung cancer. Nat. Med..

[7] Cheng Y.-H., Chen Y.-C., Lin E., Brien R., Jung S., Chen Y.-T., Lee W., Hao Z., Sahoo S., Min Kang H. (2019). Hydro-Seq enables contamination-free high-throughput single-cell RNA-sequencing for circulating tumor cells. Nat. Commun..

[8] Heitzer E., Haque I.S., Roberts C.E.S., Speicher M.R. (2019). Current and future perspectives of liquid biopsies in genomics-driven oncology. Nat. Rev. Genet..

[9] Brown N.A., Elenitoba-Johnson K.S.J. (2020). Enabling Precision Oncology Through Precision Diagnostics. Annu. Rev. Pathol..

[10] IJzerman M.J., de Boer J., Azad A., Degeling K., Geoghegan J., Hewitt C., Hollande F., Lee B., To Y.H., Tothill R.W. (2021). Towards Routine Implementation of Liquid Biopsies in Cancer Management: It Is Always Too Early, until Suddenly It Is Too Late. Diagnostics.

[11] Christensen E., Birkenkamp-Demtröder K., Sethi H., Shchegrova S., Salari R., Nordentoft I., Wu H.-T., Knudsen M., Lamy P., Lindskrog S.V. (2019). Early Detection of Metastatic Relapse and Monitoring of Therapeutic Efficacy by Ultra-Deep Sequencing of Plasma Cell-Free DNA in Patients with Urothelial Bladder Carcinoma. J. Clin. Oncol..

[12] McDonald B.R., Contente-Cuomo T., Sammut S.-J., Odenheimer-Bergman A., Ernst B., Perdigones N., Chin S.-F., Farooq M., Mejia R., Cronin P.A. (2019). Personalized circulating tumor DNA analysis to detect residual disease after neoadjuvant therapy in breast cancer. Sci. Transl. Med..

[13] Leal A., van Grieken N.C.T., Palsgrove D.N., Phallen J., Medina J.E., Hruban C., Broeckaert M.A.M., Anagnostou V., Adleff V., Bruhm D.C. (2020). White blood cell and cell-free DNA analyses for detection of residual disease in gastric cancer. Nat. Commun..

[14] Cardoso F., Paluch-Shimon S., Senkus E., Curigliano G., Aapro M.S., André F., Barrios C.H., Bergh J., Bhattacharyya G.S., Biganzoli L. (2020). 5th ESO-ESMO international consensus guidelines for advanced breast cancer (ABC 5). Ann. Oncol..

[15] Planchard D., Popat S., Kerr K., Novello S., Smit E.F., Faivre-Finn C., Mok T.S., Reck M., van Schil P.E., Hellmann M.D. (2018). Metastatic non-small cell lung cancer: ESMO Clinical Practice Guidelines for diagnosis, treatment and follow-up. Ann. Oncol..

[16] Tie J., Cohen J.D., Lo S.N., Wang Y., Li L., Christie M., Lee M., Wong R., Kosmider S., Skinner I. (2020). Prognostic significance of postsurgery circulating tumor DNA in nonmetastatic colorectal cancer: Individual patient pooled analysis of three cohort studies. Int. J. Cancer.

[17] Argilés G., Tabernero J., Labianca R., Hochhauser D., Salazar R., Iveson T., Laurent-Puig P., Quirke P., Yoshino T., Taieb J. (2020). Localised colon cancer: ESMO Clinical Practice Guidelines for diagnosis, treatment and follow-up. Ann. Oncol..

[18] Sørbye H., Dahl O. (2003). Carcinoembryonic Antigen Surge in Metastatic Colorectal Cancer Patients Responding to Oxaliplatin Combination Chemotherapy: Implications for Tumor Marker Monitoring and Guidelines. J. Clin. Oncol..

[19] Goldstein M., Mitchell E.P. (2005). Carcinoembryonic Antigen in the Staging and Follow-up of Patients with Colorectal Cancer. Cancer Investig..

[20] Holdhoff M., Schmidt K., Donehower R., Diaz L.A. (2009). Analysis of circulating tumor DNA to confirm somatic KRAS mutations. J. Natl. Cancer Inst..

[21] Diehl F., Li M., Dressman D., He Y., Shen D., Szabo S., Diaz L.A., Goodman S.N., David K.A., Juhl H. (2005). Detection and quantification of mutations in the plasma of patients with colorectal tumors. Proc. Natl. Acad. Sci. USA.

[22] Liebs S., Keilholz U., Kehler I., Schweiger C., Haybäck J., Nonnenmacher A. (2019). Detection of mutations in circulating cell-free DNA in relation to disease stage in colorectal cancer. Cancer Med..

[23] Godsey J.H., Silvestro A., Barrett J.C., Bramlett K., Chudova D., Deras I., Dickey J., Hicks J., Johann D.J., Leary R. (2020). Generic Protocols for the Analytical Validation of Next-Generation Sequencing-Based ctDNA Assays: A Joint Consensus Recommendation of the BloodPAC’s Analytical Variables Working Group. Clin. Chem..

[24] NCCLS (2004). Protocols for Determination of Limits of Detection and Limits of Quantitation Guideline: Approved Guideline.

[25] Wittekind C., Meyer H.-J. (2010). TNM Klassifikation Maligner Tumoren.

[26] Midic D., Rinke J., Perner F., Müller V., Hinze A., Pester F., Landschulze J., Ernst J., Gruhn B., Matziolis G. (2020). Prevalence and dynamics of clonal hematopoiesis caused by leukemia-associated mutations in elderly individuals without hematologic disorders. Leukemia.

[27] Hallermayr A., Benet-Pagès A., Steinke-Lange V., Mansmann U., Rentsch M., Holinski-Feder E., Pickl J.M.A. (2021). Liquid Biopsy Hotspot Variant Assays: Analytical Validation for Application in Residual Disease Detection and Treatment Monitoring. Clin. Chem..

[28] Flamini E., Mercatali L., Nanni O., Calistri D., Nunziatini R., Zoli W., Rosetti P., Gardini N., Lattuneddu A., Verdecchia G.M. (2006). Free DNA and carcinoembryonic antigen serum levels: An important combination for diagnosis of colorectal cancer. Clin. Cancer Res..

[29] Czeiger D., Shaked G., Eini H., Vered I., Belochitski O., Avriel A., Ariad S., Douvdevani A. (2011). Measurement of circulating cell-free DNA levels by a new simple fluorescent test in patients with primary colorectal cancer. Am. J. Clin. Pathol..

[30] Berger A.W., Schwerdel D., Welz H., Marienfeld R., Schmidt S.A., Kleger A., Ettrich T.J., Seufferlein T. (2017). Treatment monitoring in metastatic colorectal cancer patients by quantification and KRAS genotyping of circulating cell-free DNA. PLoS ONE.

[31] Garlan F., Laurent-Puig P., Sefrioui D., Siauve N., Didelot A., Sarafan-Vasseur N., Michel P., Perkins G., Mulot C., Blons H. (2017). Early Evaluation of Circulating Tumor DNA as Marker of Therapeutic Efficacy in Metastatic Colorectal Cancer Patients (PLACOL Study). Clin. Cancer Res..

[32] Hu Y., Ulrich B.C., Supplee J., Kuang Y., Lizotte P.H., Feeney N.B., Guibert N.M., Awad M.M., Wong K.-K., Jänne P.A. (2018). False-Positive Plasma Genotyping Due to Clonal Hematopoiesis. Clin. Cancer Res..

[33] Konishi T., Shimada Y., Hsu M., Tufts L., Jimenez-Rodriguez R., Cercek A., Yaeger R., Saltz L., Smith J.J., Nash G.M. (2018). Association of Preoperative and Postoperative Serum Carcinoembryonic Antigen and Colon Cancer Outcome. JAMA Oncol..

[34] Weaver C.H. Understanding The CEA Test in Colon Cancer. https://news.cancerconnect.com/colon-cancer/understanding-the-cea-test-in-colon-cancer-2yuVIMszUkideV0m-kWBwA.

[35] Larribère L., Martens U.M. (2021). Advantages and Challenges of Using ctDNA NGS to Assess the Presence of Minimal Residual Disease (MRD) in Solid Tumors. Cancers.

[36] Reinert T., Henriksen T.V., Christensen E., Sharma S., Salari R., Sethi H., Knudsen M., Nordentoft I., Wu H.-T., Tin A.S. (2019). Analysis of Plasma Cell-Free DNA by Ultradeep Sequencing in Patients with Stages I to III Colorectal Cancer. JAMA Oncol..

[37] Wang Y., Li L., Cohen J.D., Kinde I., Ptak J., Popoli M., Schaefer J., Silliman N., Dobbyn L., Tie J. (2019). Prognostic Potential of Circulating Tumor DNA Measurement in Postoperative Surveillance of Nonmetastatic Colorectal Cancer. JAMA Oncol..

[38] Jeffery M., Hickey B.E., Hider P.N., See A.M. (2016). Follow-up strategies for patients treated for non-metastatic colorectal cancer. Cochrane Database Syst. Rev..

[39] Nicholson B.D., Shinkins B., Pathiraja I., Roberts N.W., James T.J., Mallett S., Perera R., Primrose J.N., Mant D. (2015). Blood CEA levels for detecting recurrent colorectal cancer. Cochrane Database Syst. Rev..

[40] Park G., Park J.K., Son D.-S., Shin S.-H., Kim Y.J., Jeon H.-J., Lee J., Park W.-Y., Lee K.H., Park D. (2018). Utility of targeted deep sequencing for detecting circulating tumor DNA in pancreatic cancer patients. Sci. Rep..

[41] Murahashi S., Akiyoshi T., Sano T., Fukunaga Y., Noda T., Ueno M., Zembutsu H. (2020). Serial circulating tumour DNA analysis for locally advanced rectal cancer treated with preoperative therapy: Prediction of pathological response and postoperative recurrence. Br. J. Cancer.

[42] COSMIC Cancer Browser: Large Intestine—Carcinoma—Adenocarcinoma Genes. https://cancer.sanger.ac.uk/cosmic/browse/tissue?wgs=off&sn=large_intestine&ss=all&hn=carcinoma&sh=adenocarcinoma&in=t&src=tissue&all_data=n.

[43] van Cutsem E., Cervantes A., Adam R., Sobrero A., van Krieken J.H., Aderka D., Aranda Aguilar E., Bardelli A., Benson A., Bodoky G. (2016). ESMO consensus guidelines for the management of patients with metastatic colorectal cancer. Ann. Oncol..

[44] Huang X., Gao P., Song Y., Sun J., Chen X., Zhao J., Liu J., Xu H., Wang Z. (2014). Relationship between circulating tumor cells and tumor response in colorectal cancer patients treated with chemotherapy: A meta-analysis. BMC Cancer.

[45] Wang L., Zhou S., Zhang W., Wang J., Wang M., Hu X., Liu F., Zhang Y., Jiang B., Yuan H. (2019). Circulating tumor cells as an independent prognostic factor in advanced colorectal cancer: A retrospective study in 121 patients. Int. J. Colorectal Dis..

[46] Ding Y., Li W., Wang K., Xu C., Hao M., Ding L. (2020). Perspectives of the Application of Liquid Biopsy in Colorectal Cancer. Biomed Res. Int..

[47] Vacante M., Ciuni R., Basile F., Biondi A. (2020). The Liquid Biopsy in the Management of Colorectal Cancer: An Overview. Biomedicines.

[48] Lambros M.B., Gil V.S., Crespo M., Fontes M.S., Neves R.N., Mahra N., Fowler G., Ebbs B., Flohr P., Seed G. (2017). Abstract 993: Diagnostic leukapheresis (DLA): Molecular characterisation and organoid culture of circulating tumor cells (CTC) from metastatic castration resistant prostate cancer (mCRPC). Cancer Res..

[49] Schou J.V., Rossi S., Jensen B.V., Nielsen D.L., Pfeiffer P., Høgdall E., Yilmaz M., Tejpar S., Delorenzi M., Kruhøffer M. (2014). miR-345 in metastatic colorectal cancer: A non-invasive biomarker for clinical outcome in non-KRAS mutant patients treated with 3rd line cetuximab and irinotecan. PLoS ONE.

[50] Tsukamoto M., Iinuma H., Yagi T., Matsuda K., Hashiguchi Y. (2017). Circulating Exosomal MicroRNA-21 as a Biomarker in Each Tumor Stage of Colorectal Cancer. Oncology.

[51] Fu F., Jiang W., Zhou L., Chen Z. (2018). Circulating Exosomal miR-17-5p and miR-92a-3p Predict Pathologic Stage and Grade of Colorectal Cancer. Transl. Oncol..

[52] Jin G., Liu Y., Zhang J., Bian Z., Yao S., Fei B., Zhou L., Yin Y., Huang Z. (2019). A panel of serum exosomal microRNAs as predictive markers for chemoresistance in advanced colorectal cancer. Cancer Chemother. Pharmacol..

