# Supplementary material for: Clinical Validity of Circulating Tumor DNA as Prognostic and Predictive Marker for Personalized Colorectal Cancer Patient Management
Source: Cancers (Basel). 2022 Feb 8;14(3):851. doi: 10.3390/cancers14030851 (PMC8834623; doi:10.3390/cancers14030851)
Supplement: Supplementary file 1 [file cancers-14-00851-s001.zip › Supplement.pdf]

Supplementary Methods

Clinical sample collection

Generally CtDNA samples were collected at routinely scheduled follow-up examinations of CRC patients until death or patient withdrawal from the study.

Tumor tissue was retrieved during diagnostic colonoscopy or primary surgery. Clinical data were collected for all patients throughout the course of disease (Supplemental Table S1). ctDNA samples were retrospectively analyzed without prior knowledge of patient's outcome. The study was approved by the ethics commission of the Bavarian Medical Association (No. 17059) and is registered with the German registry for clinical trials (trial registration ID: DRKS00012890). Neither clinicians nor patients were informed about the results. All participants provided informed written consent prior to blood and tissue specimen collection.

For healthy controls, cfDNA from plasma of healthy individuals was used.

Control samples

For positive controls, *in vitro* dilutions containing mutant variant allele frequencies (VAFs) ranging from 1% to 5% were generated by spiking genomic DNA (gDNA) from cell lines into gDNA from whole blood of healthy controls. For *BRAF* p.V600E variant, gDNA from SK-MEL-28 cell line (#Cat 300337GD1) and for *KRAS* p.G12S variant, gDNA from A-549 cell line (#Cat300114GD1) were purchased from CLS (Eppelheim, Germany). For wild-type (WT) controls, gDNA from whole blood of healthy individuals was used.

Sample preparation

Blood samples were collected in Cell-Free DNA BCT (STRECK, La Vista, Nebraska, USA, #Cat 230244) tubes. Plasma was separated from whole blood by centrifugation at 1,600 x g for 10 min at room-temperature (RT) and was transferred to a new 15 ml reaction tube, followed by two centrifugations at 16,000 x g for 10 min at 4°C. Buffy coat was transferred to a 1.5 ml reaction tube followed by two times washing with 1x PBS. Plasma and buffy coat were stored at -80°C until further sample processing.

cfDNA extraction

Cell-free DNA from 2 to 7.5 ml plasma was isolated using the QIAamp circulating nucleic acid kit (Qiagen, Hilden, Germany, #Cat 55114). All buffer volumes were adjusted to the respective plasma volumes. All membrane washing steps were performed twice. cfDNA concentration was quantified using the High

Sensitivity NGS Fragment Analysis Kit (Agilent, Santa Clara, California, USA, #DNF-474-0500) on the Fragment Analyzer system (Agilent).

gDNA extraction

Genomic DNA from whole blood was extracted on a Biomek® FX system (Beckman Coulter, Brea, California, USA) using the NucleoMag® Blood 3 ml Kit (Machery-Nagel, Düren, Germany, #REF 744502.1) as by manufacturer's instructions. gDNA from formalin-fixed paraffin embedded (FFPE) tissue was extracted using the QIAamp DNA FFPE tissue kit (Qiagen, #Cat 56404) as by manufacturers' instructions. gDNA from buffy coat was extracted using the FlexiGene DNA Kit (Qiagen, #Cat 51206). All centrifugation steps were performed as by manufacturers' instructions, with the following adjustments. Following complete homogenization in buffer FG2 containing QIAGEN Protease samples were incubated for 2 h at 65°C. DNA was precipitated by adding 600 µl of 100% isopropanol. After centrifugation, DNA pellet was washed with 300 µl of 70% ethanol. DNA was eluted after air drying the pellet, in 30 – 200 µl FG3 buffer, by incubation on a heating block for 75 min at 65°C followed by overnight incubation at 37°C and 950 rpm. Concentration of gDNA was determined using a Slide-200 on an Xpose – Benchtop Spectrophotometer (Trinean, Pleasanton, California, USA).

ddPCR protocol

DNA was mixed with 10 µl of ddPCR™ Supermix for Probes (no dUTPs) (Bio-Rad, #Cat 1863023) and 1 µl of the primer/probe mixture. For gDNA from whole blood, cell lines or tumor specimens, 5 µl of extracted DNA at a concentration of 4-6 ng/µl were used in single reactions. For cfDNA from plasma samples, 1 µl, 5 µl or 8 µl of extracted DNA at concentrations of >15 ng/µl, between 2 ng/µl and 15 ng/µl, and between 0.6 ng/µl and 2 ng/µl respectively, were used in three replicates. 70 µl of Droplet Generation Oil for Probes (Bio-Rad, #Cat 1863005) was added to the reaction mix for droplet generation in a DG8 cartridge. Droplets were transferred into a 96-well plate (Bio-Rad, #Cat 12001925) and thermal cycled (Eppendorf, Hamburg, Germany, Mastercycler pro): 10 minutes at 95°C, 40 cycles of 94°C for 30 s, 55°C for 1 minute followed by 98°C for 10 minutes (Ramp Rate 2°C/sec).

Supplementary Data

Study cohort

Supplementary Table S1 Baseline characteristics of study participants

Characteristics	Healthy	CRC	Stage I	Stage II	Stage III	Stage IV
**Number of individuals**	**80**	**29**	**4**	**8**	**5**	**12**
**Number of plasma samples**						
Total	80	132	16	43	20	53
Median	1	4	4.5	4	5	2
Range	1	1-15	2-5	2-15	1-7	1-15
**Baseline**						
ctDNA	20	22	4	8	4	6
cfDNA	60	27	4	8	5	11
CEA	NA	21	4	8	4	5
**Follow-up**						
ctDNA	NA	102	12	35	15	40
cfDNA	NA	101	12	35	15	39
CEA	NA	99	12	35	15	37
**Follow-up time (months)**						
Median	NA	3.9	11.9	7.6	10.8	0.3
Range	NA	0-27.4	0.1-12.4	0.2-27.4	0-11.9	0-17.0
**Residual disease / Recurrence**	NA	18	4	8	4	2
**Monitoring**	NA	9	0	1	3	5
**Tissue genetic status**						
KRAS p.G12/p.G13	NA	12	2	4	3	3
BRAF p.V600E	NA	10	2	4	1	3
**Age at enrollment**						
Median	31	73	69	69.5	78	71
Range	19-73	39-87	54-86	61-85	75-86	39-87
**Sex**						
Female	43	13	2	4	1	6
Male	35	16	2	4	4	6

Residual disease

LB-CRC-03

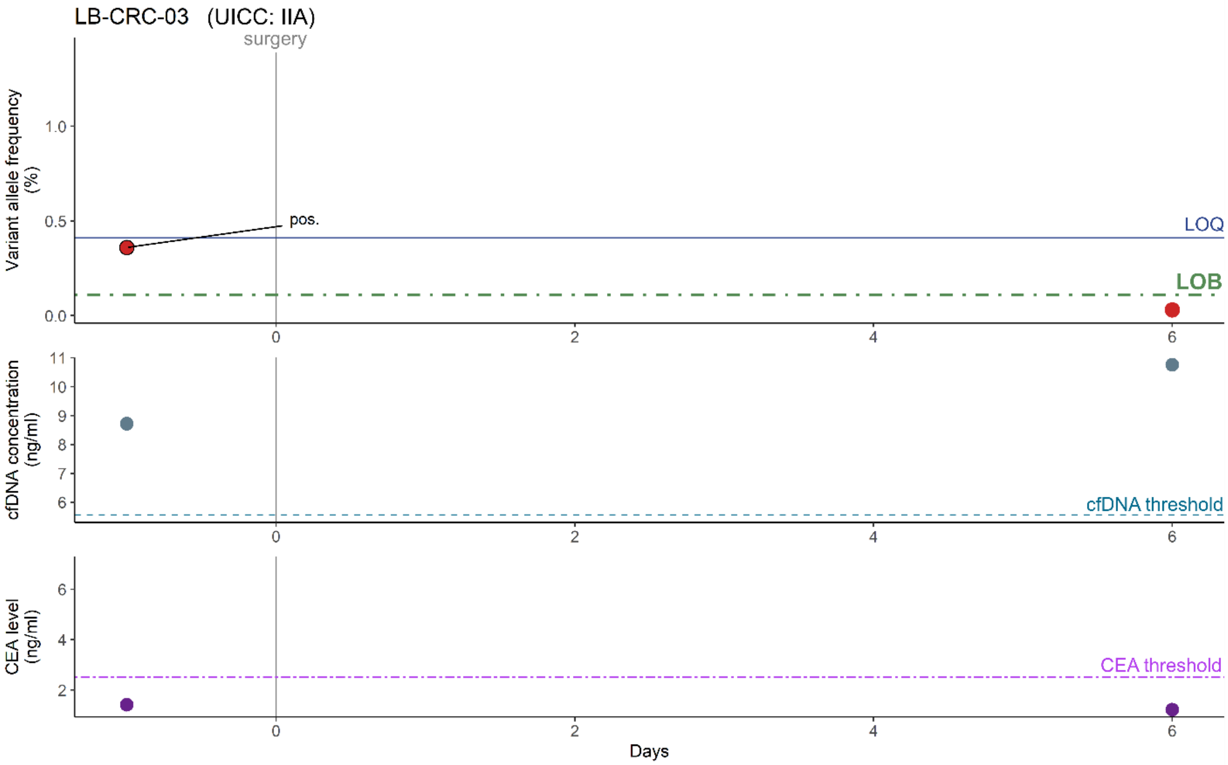

Supplementary Figure S1 Residual disease detection in patient LB-CRC-03.

Patient LB-CRC-03 was diagnosed with stage IIA CRC. ctDNA was present at baseline (>LOB) and absent 6 days after surgery (<LOB).

LB-CRC-05

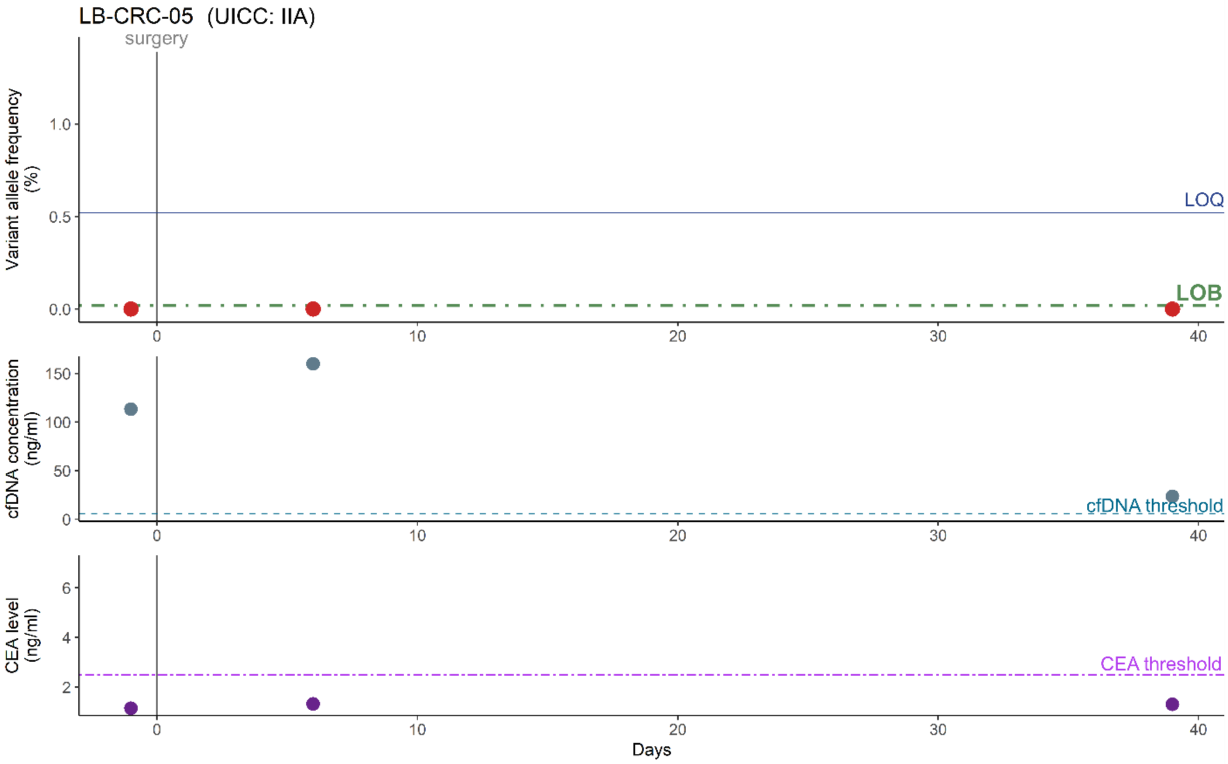

Supplementary Figure S2 Residual disease detection in patient LB-CRC-05.

Patient LB-CRC-05 was diagnosed with stage IIA CRC. ctDNA was absent at baseline as well as 7 and 39 days after surgery (<LOB).

LB-CRC-06

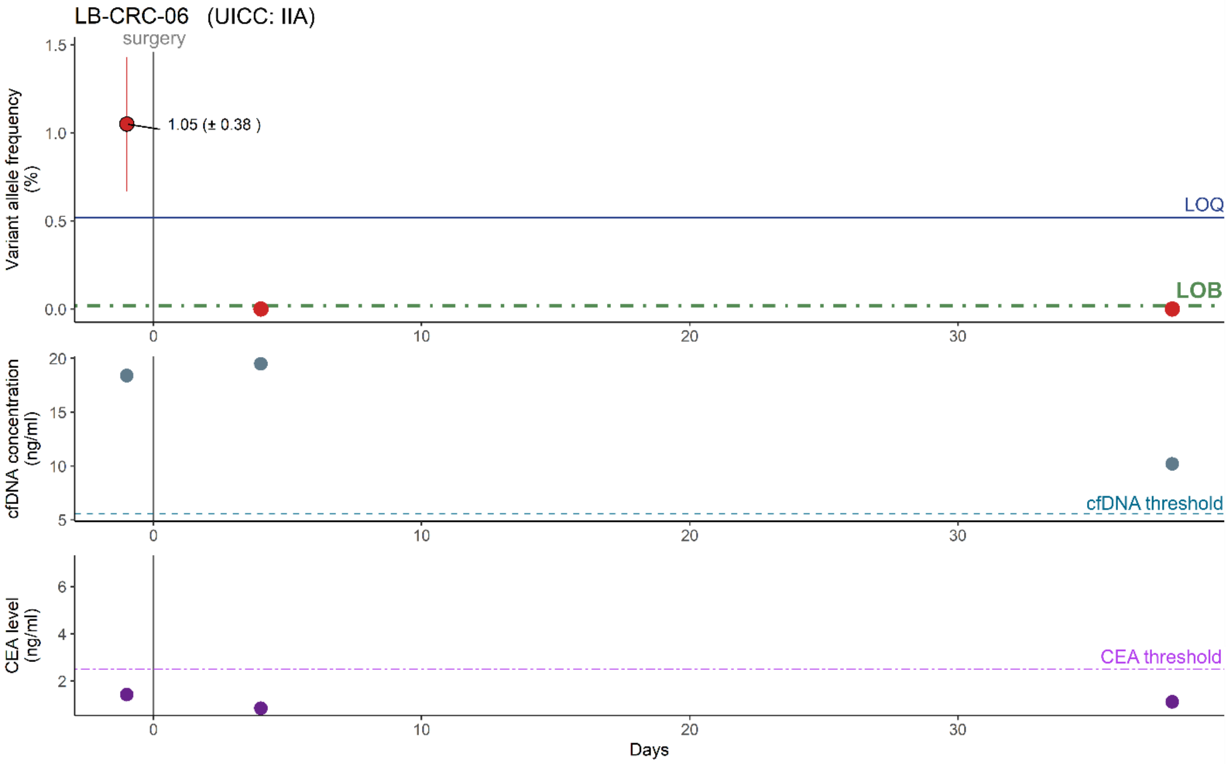

Supplementary Figure S3 Residual disease detection in patient LB-CRC-06.

Patient LB-CRC-06 was diagnosed with stage IIA CRC. ctDNA was present at baseline with a mutant VAF of 1.05% (>LOQ) and absent 4 and 38 after surgery (>LOB).

LB-CRC-07

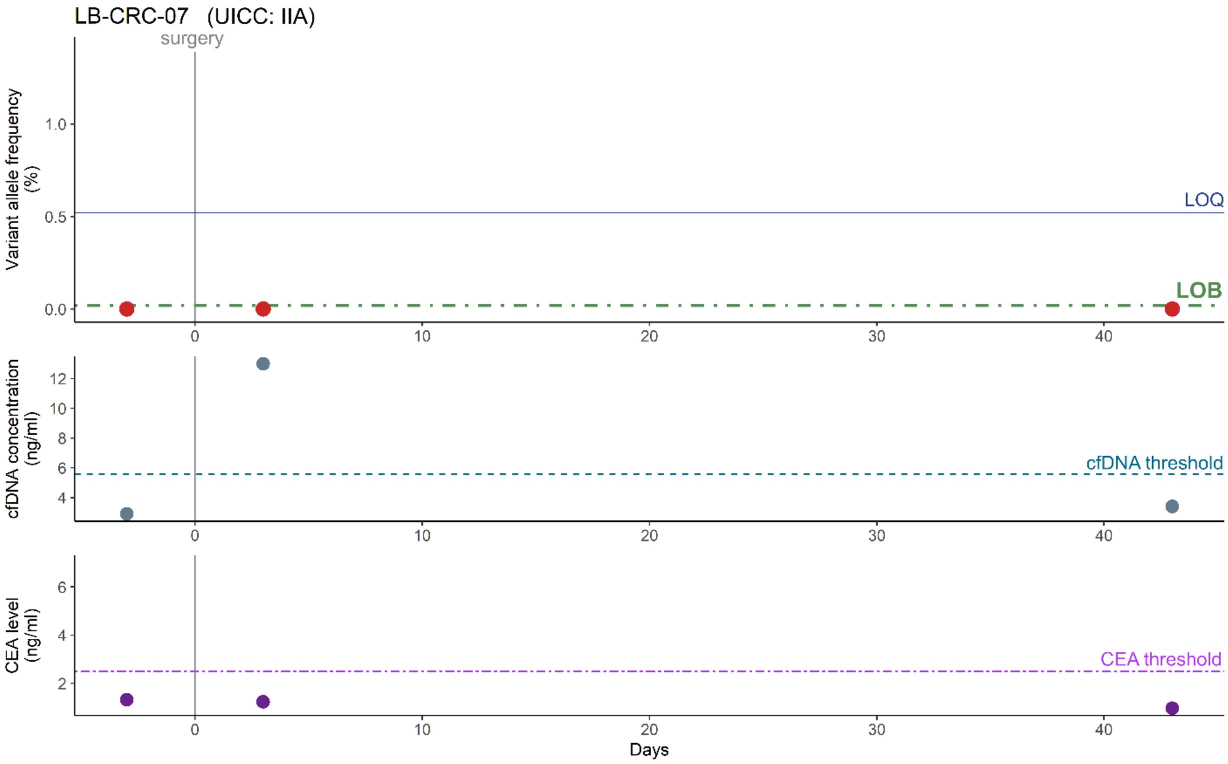

Supplementary Figure S4 Residual disease detection in patient LB-CRC-07.

Patient LB-CRC-07 was diagnosed with stage IIA CRC. ctDNA was absent at baseline as well as 4 and 43 days after surgery (<LOB).

LB-CRC-09

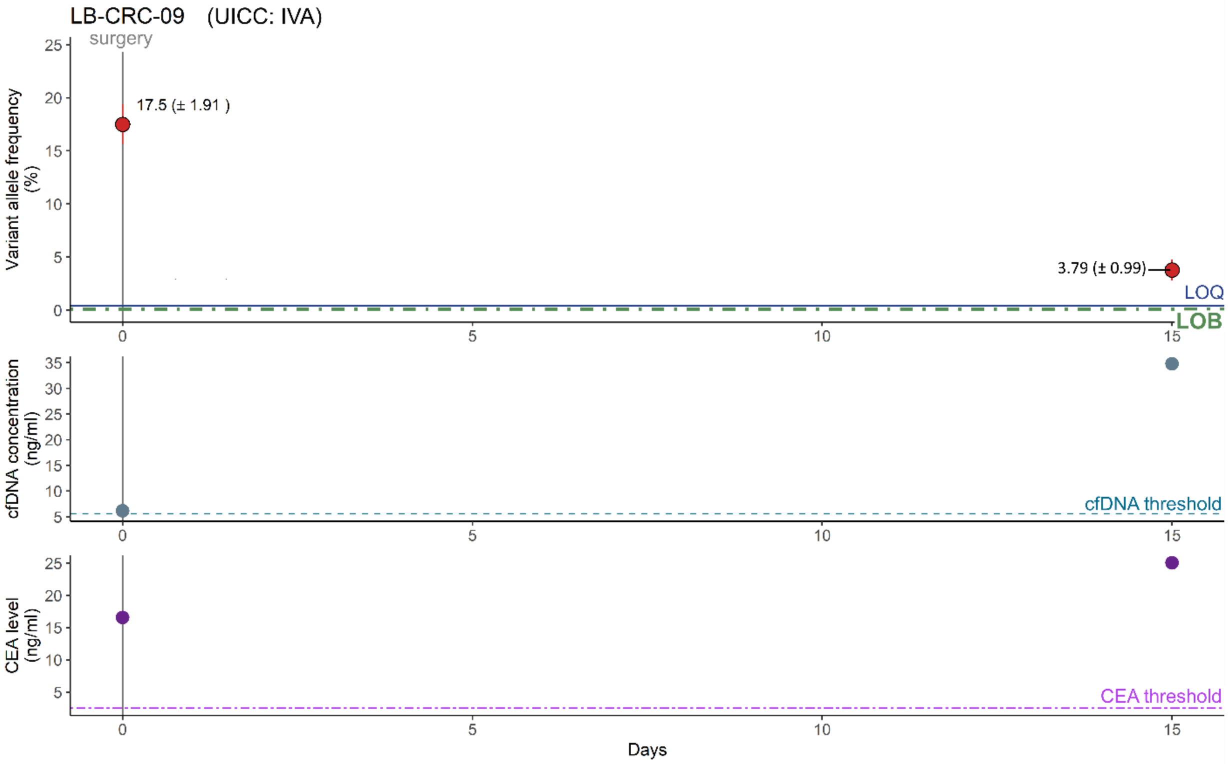

Supplementary Figure S5 Residual disease detection in patient LB-CRC-09.

Patient LB-CRC-09 was diagnosed with stage IVA CRC. ctDNA was present at baseline with a mutant VAF of 17.5% which decreased to 3.79% 15 days after surgery (>LOQ).

LB-CRC-10

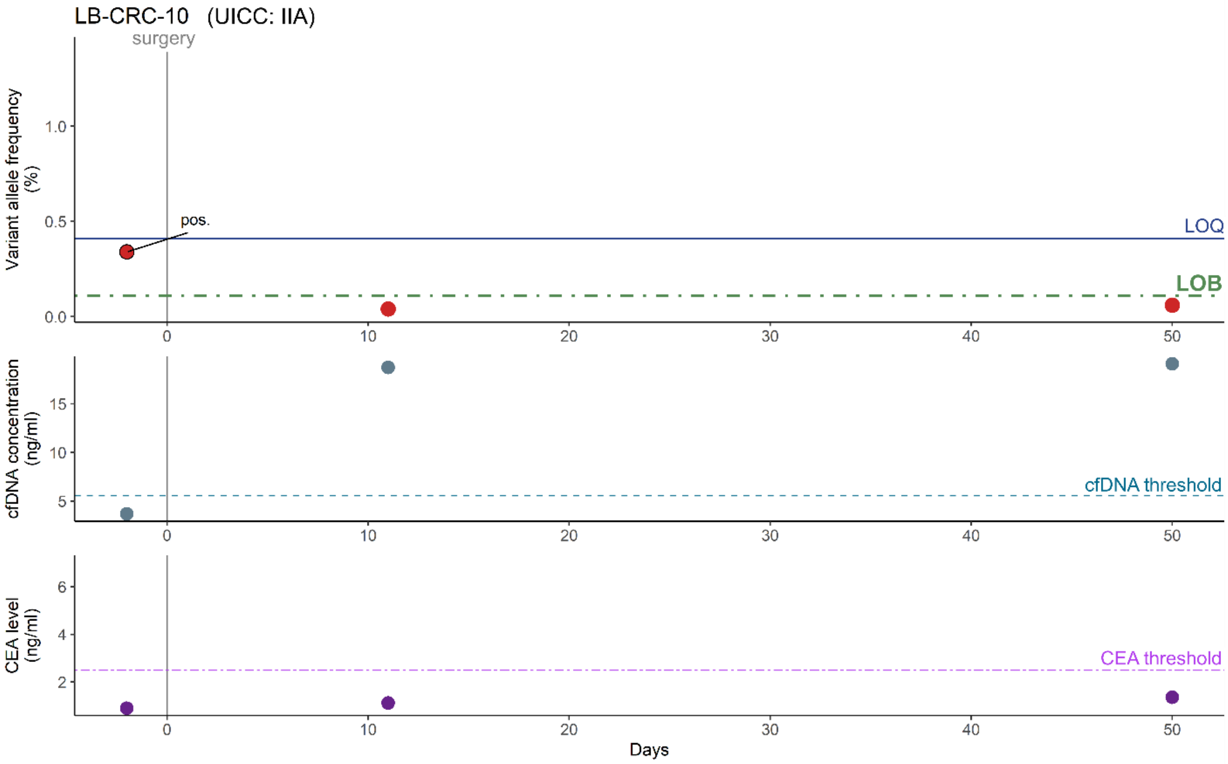

Supplementary Figure S6 Residual disease detection in patient LB-CRC-10.

Patient LB-CRC-10 was diagnosed with stage IIA CRC. ctDNA was present at baseline (>LOB) and absent 11 and 50 days after surgery (<LOB).

LB-CRC-11

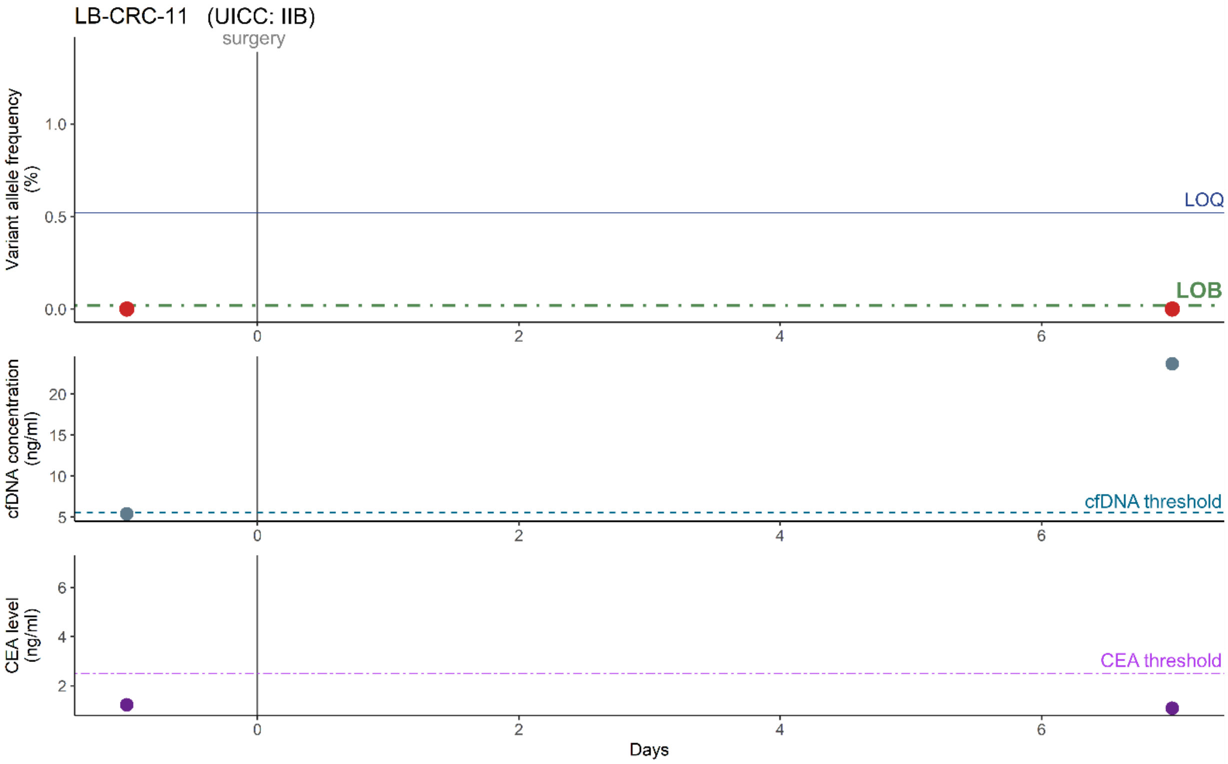

Supplementary Figure S7 Residual disease detection in patient LB-CRC-11.

Patient LB-CRC-11 was diagnosed with stage IIB CRC. ctDNA was absent at baseline as well as 7 days after surgery (<LOB).

LB-CRC-14

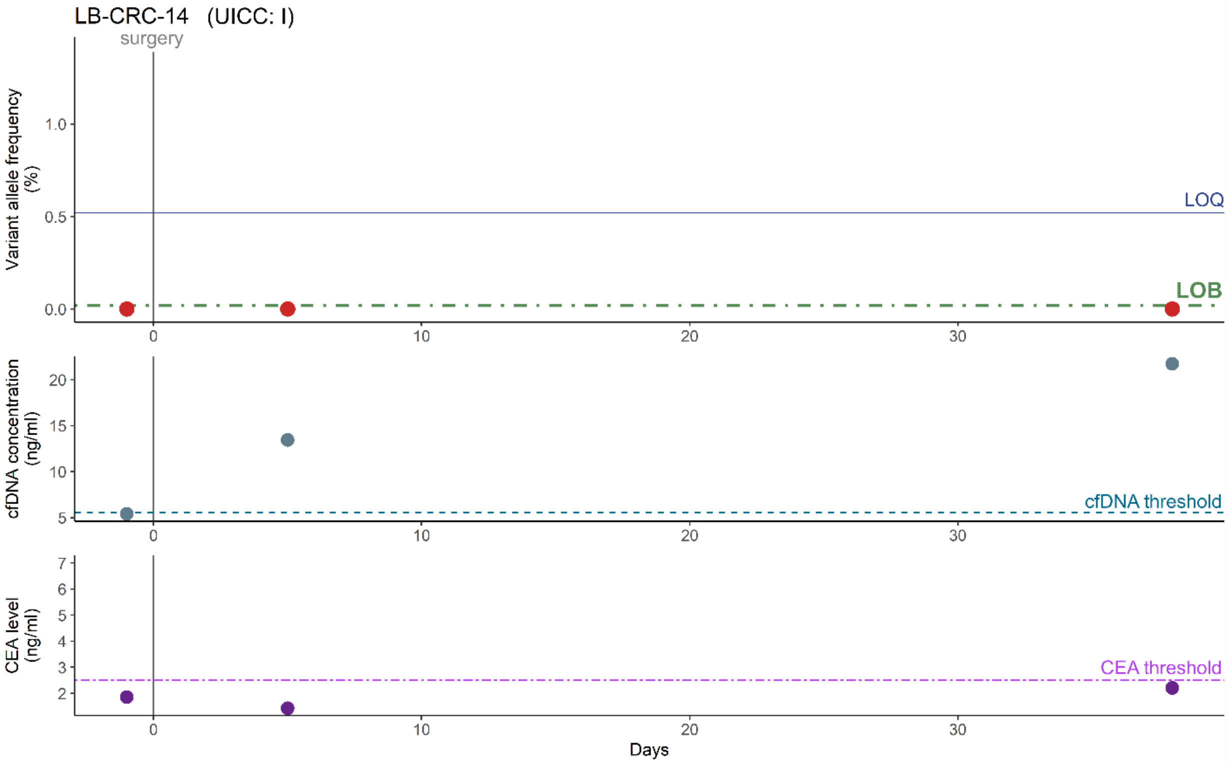

Supplementary Figure S8 Residual disease detection in patient LB-CRC-14.

Patient LB-CRC-14 was diagnosed with stage I CRC. ctDNA was absent at baseline as well as 5 and 38 days after surgery (<LOB).

LB-CRC-18

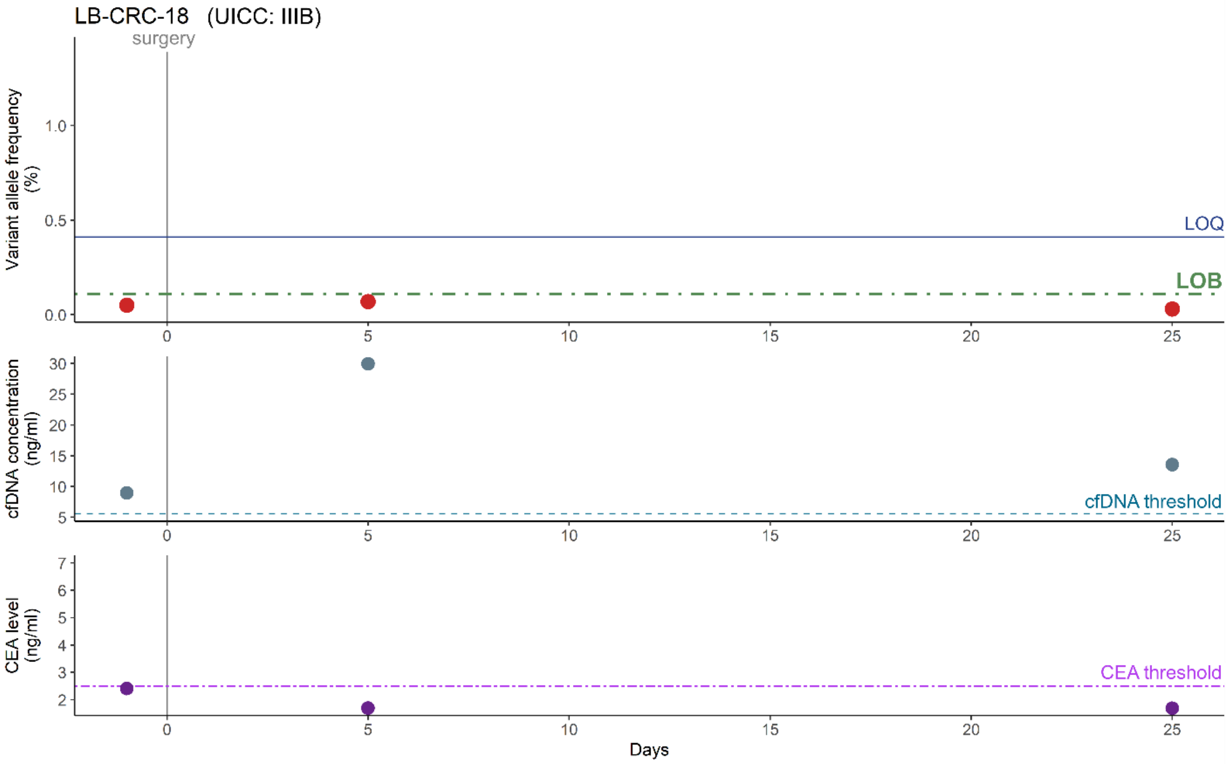

Supplementary Figure S9 Residual disease detection in patient LB-CRC-18.

Patient LB-CRC-18 was diagnosed with stage IIIB CRC. ctDNA was absent at baseline as well as 5 and 25 days after surgery (<LOB).

LB-CRC-19

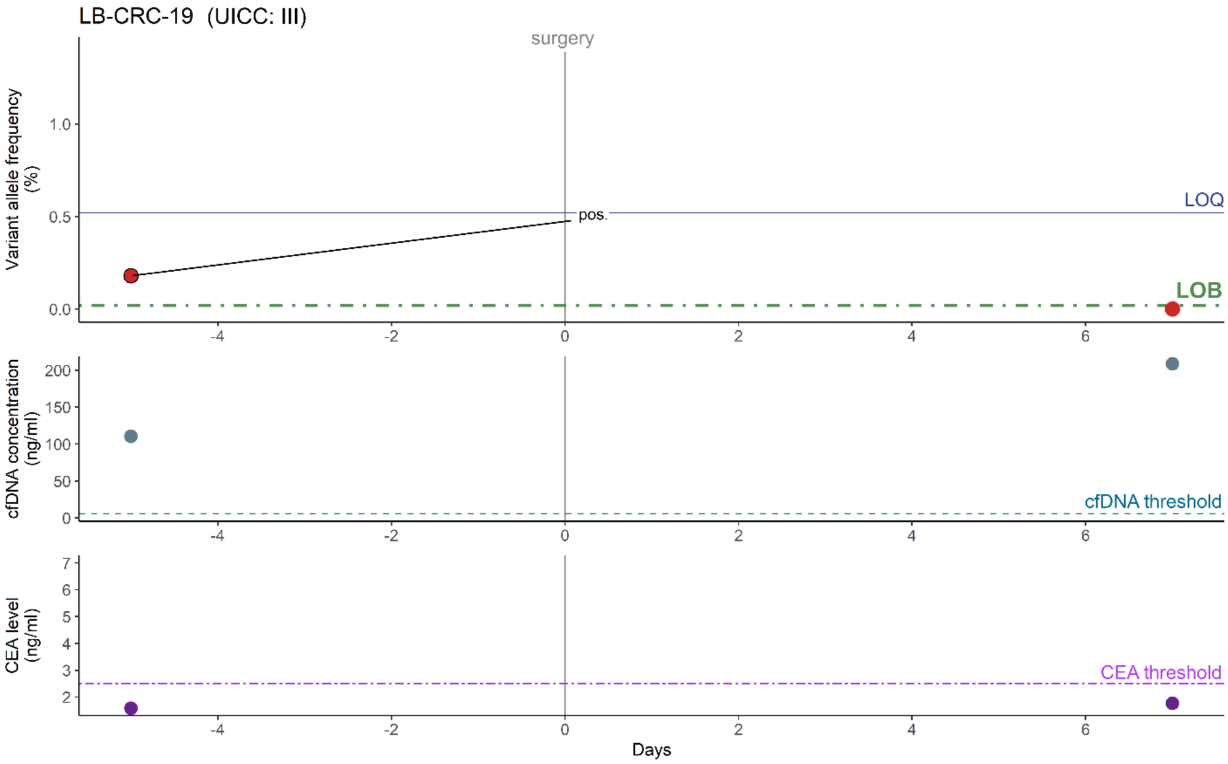

Supplementary Figure S10 Residual disease detection in patient LB-CRC-19.

Patient LB-CRC-19 was diagnosed with stage III CRC. ctDNA was present at baseline (>LOB) and absent 7 days after surgery (<LOB).

LB-CRC-23

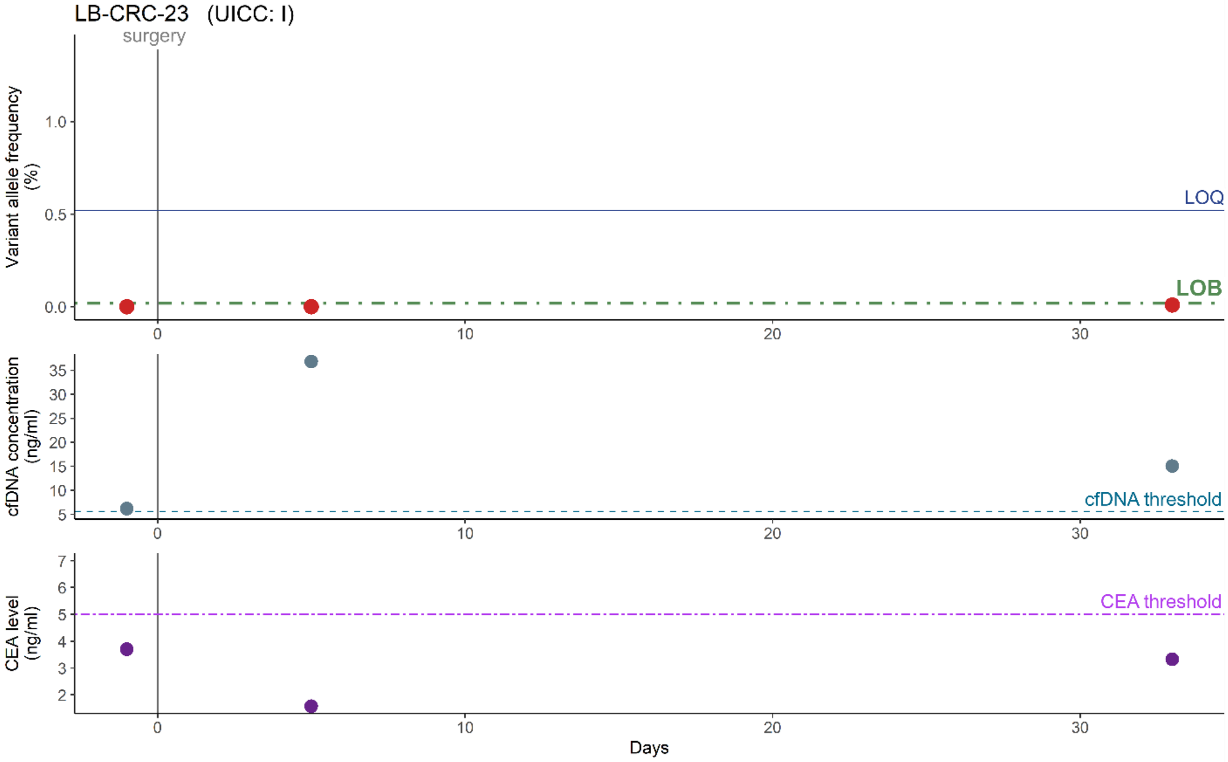

Supplementary Figure S11 Residual disease detection in patient LB-CRC-23.

Patient LB-CRC-23 was diagnosed with stage I CRC. ctDNA was absent at baseline as well as 5 and 22 days after surgery (<LOB).

LB-CRC-24

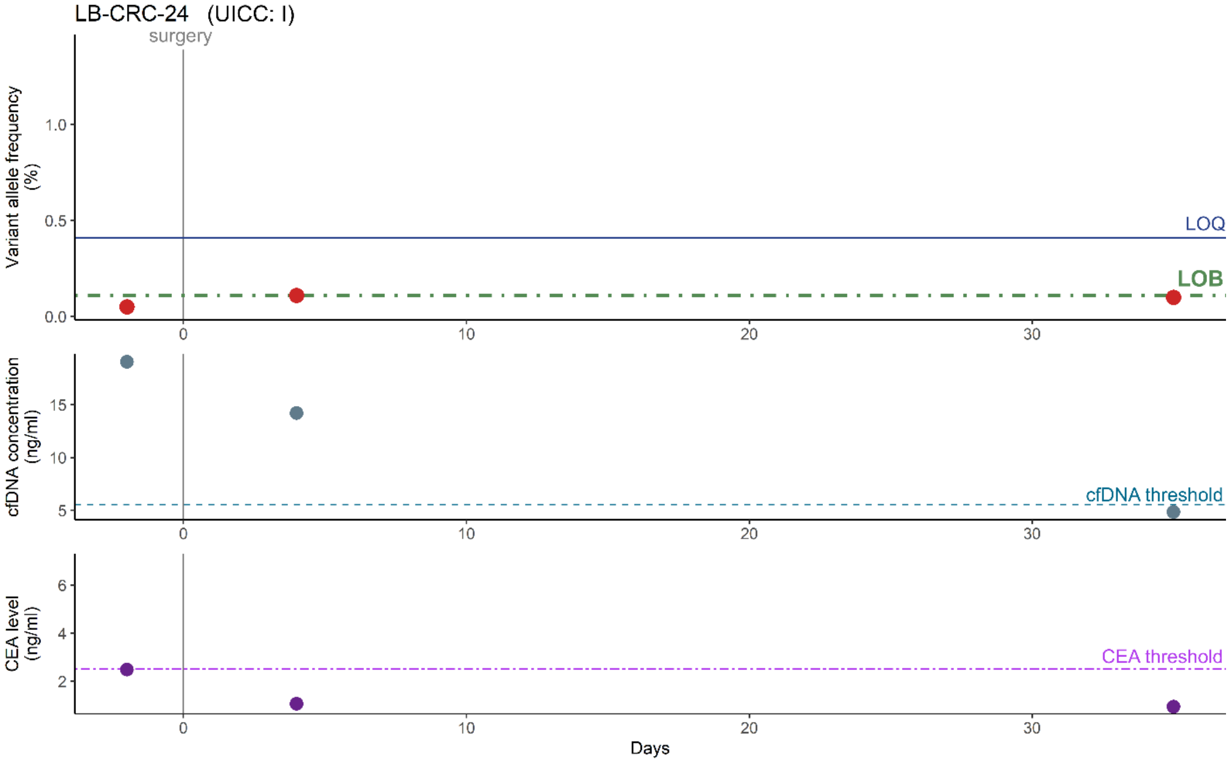

Supplementary Figure S12 Residual disease detection in patient LB-CRC-24.

Patient LB-CRC-24 was diagnosed with stage I CRC. ctDNA was absent at baseline as well as 4 and 35 days after surgery (<LOB).

LB-CRC-25

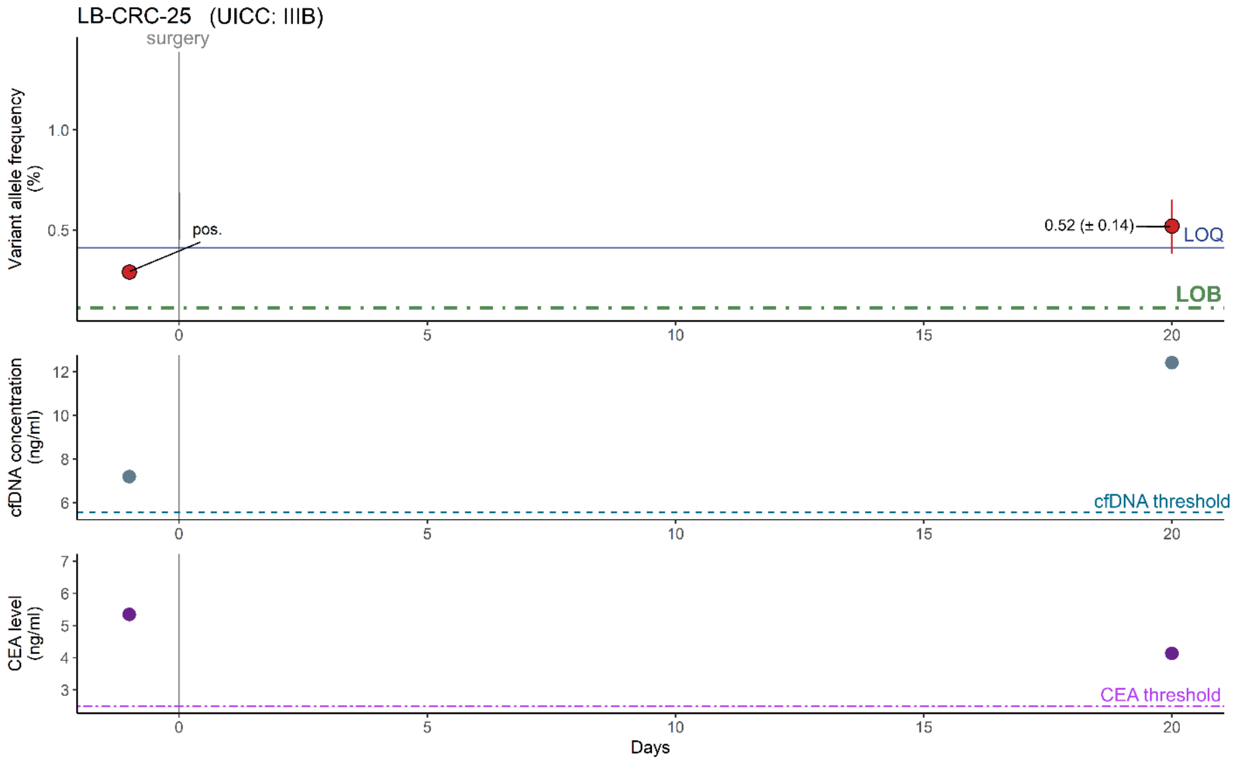

Supplementary Figure S13 Residual disease detection in patient LB-CRC-25.

Patient LB-CRC-25 was diagnosed with stage IIIB CRC. ctDNA was present at baseline(>LOB) and 20 days after surgery with a mutant VAF of 0.52% (>LOQ).

LB-CRC-29

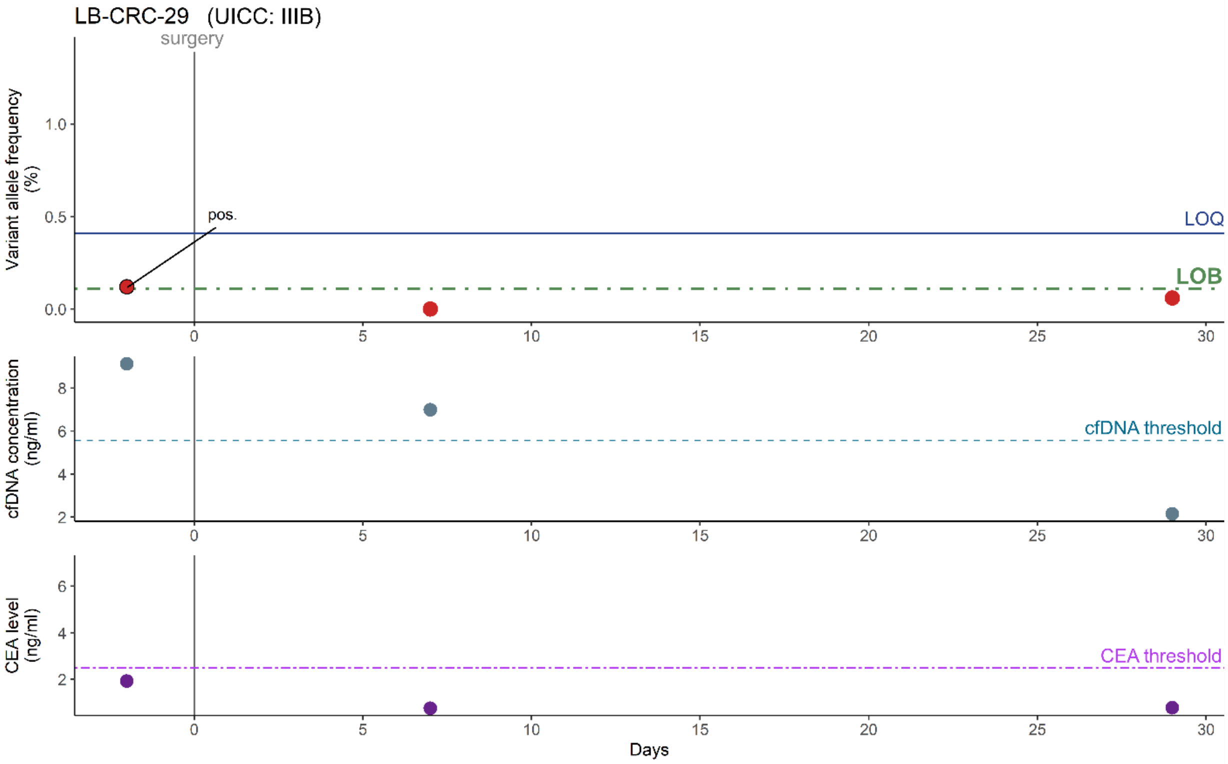

Supplementary Figure S14 Residual disease detection in patient LB-CRC-29.

Patient LB-CRC-29 was diagnosed with stage IIIB CRC. ctDNA was present at baseline (>LOB) and absent 7 and 29 days after surgery (<LOB).

LB-CRC-37

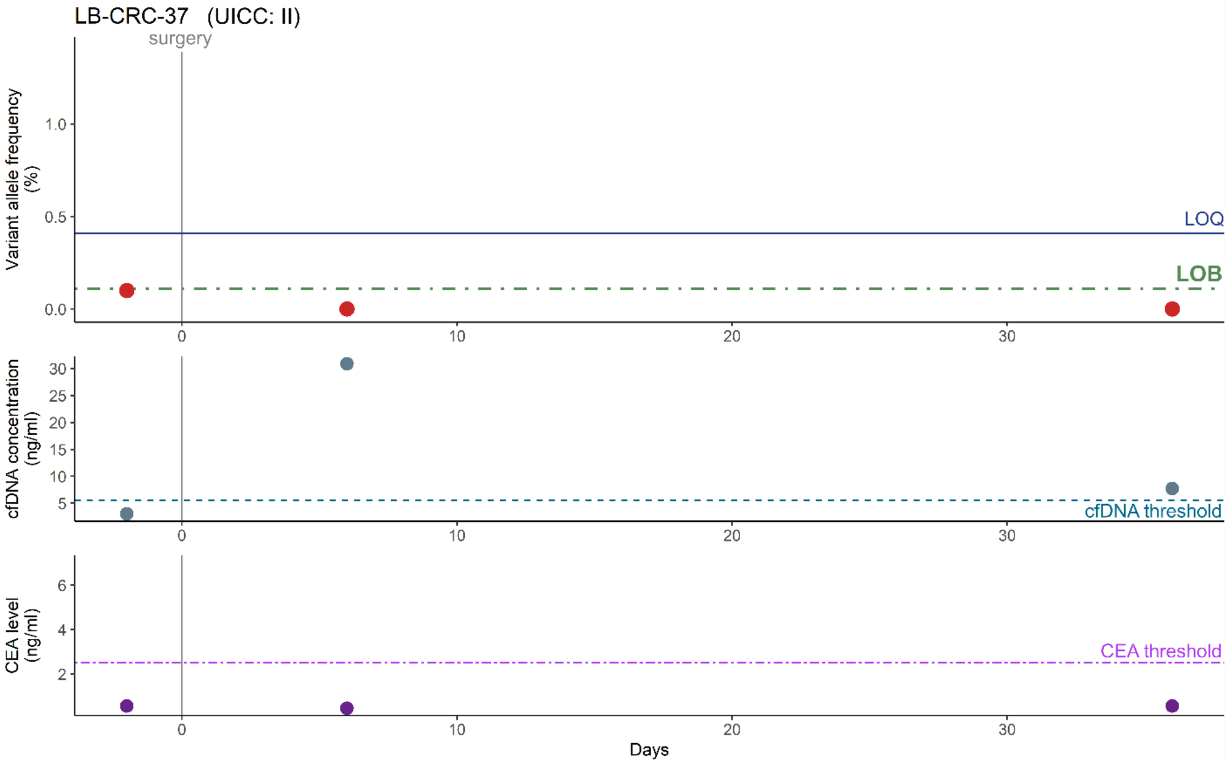

Supplementary Figure S15 Residual disease detection in patient LB-CRC-37.

Patient LB-CRC-37 was diagnosed with stage II CRC. ctDNA was absent at baseline as well as 7 and 36 days after surgery (<LOB)..

LB-CRC-38

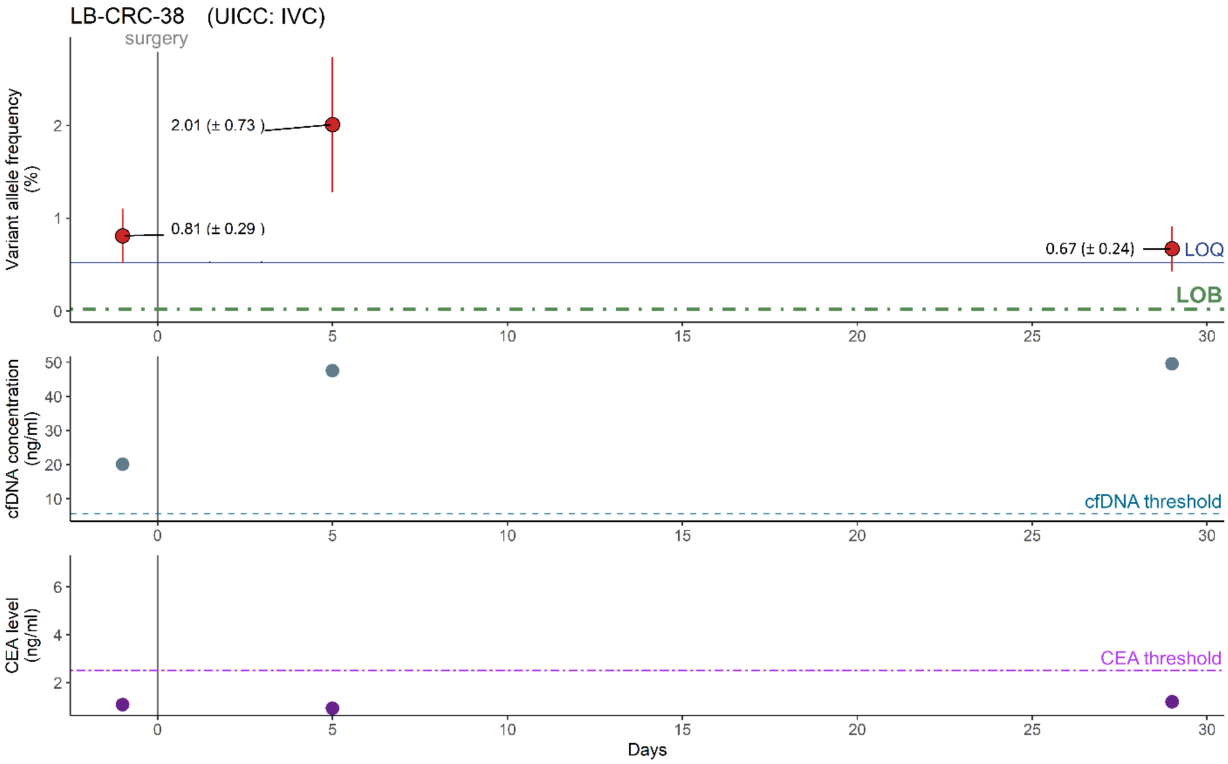

Supplementary Figure S16 Residual disease detection in patient LB-CRC-38.

Patient LB-CRC-38 was diagnosed with stage IVC CRC. ctDNA was present at baseline with a mutant VAF of 0.81% and still was in the quantifiable range 5 and 29 days after surgery (>LOQ).

LB-CRC-40

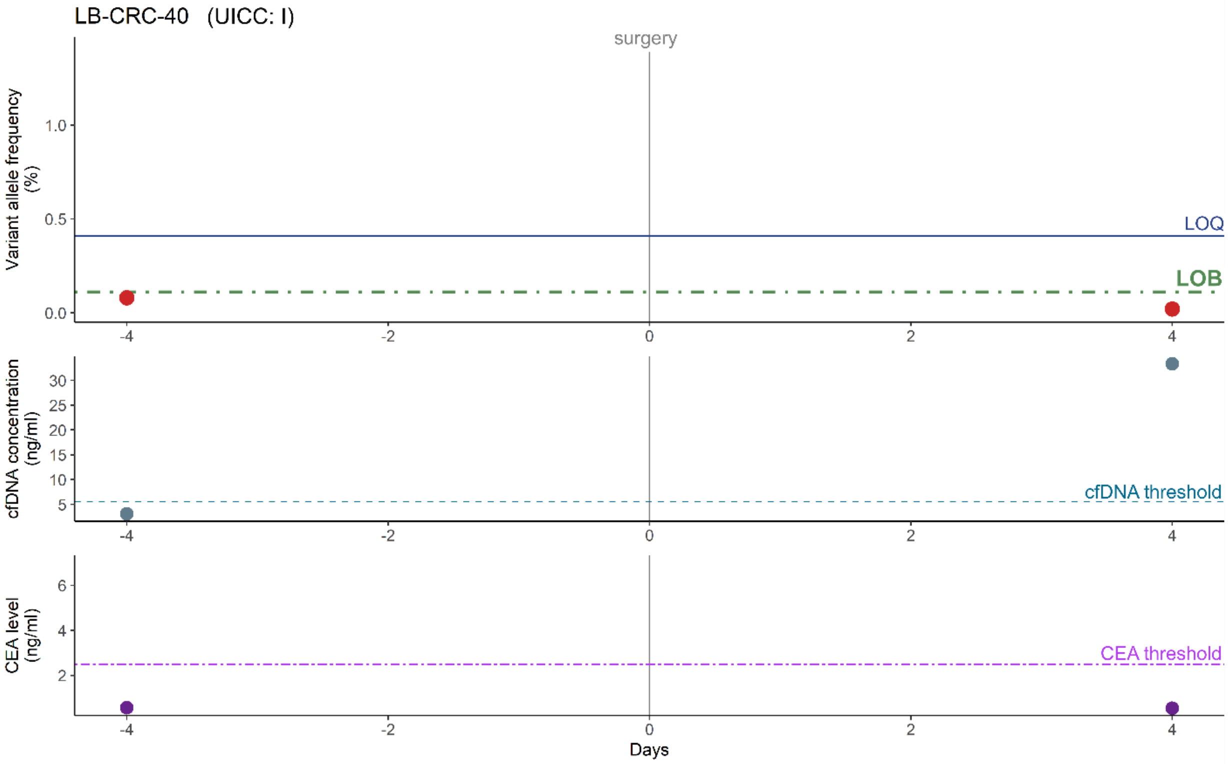

Supplementary Figure S17 Residual disease detection in patient LB-CRC-40.

Patient LB-CRC-40 was diagnosed with stage I CRC. ctDNA was absent at baseline as well as 4 days after surgery (<LOB).

LB-CRC-41

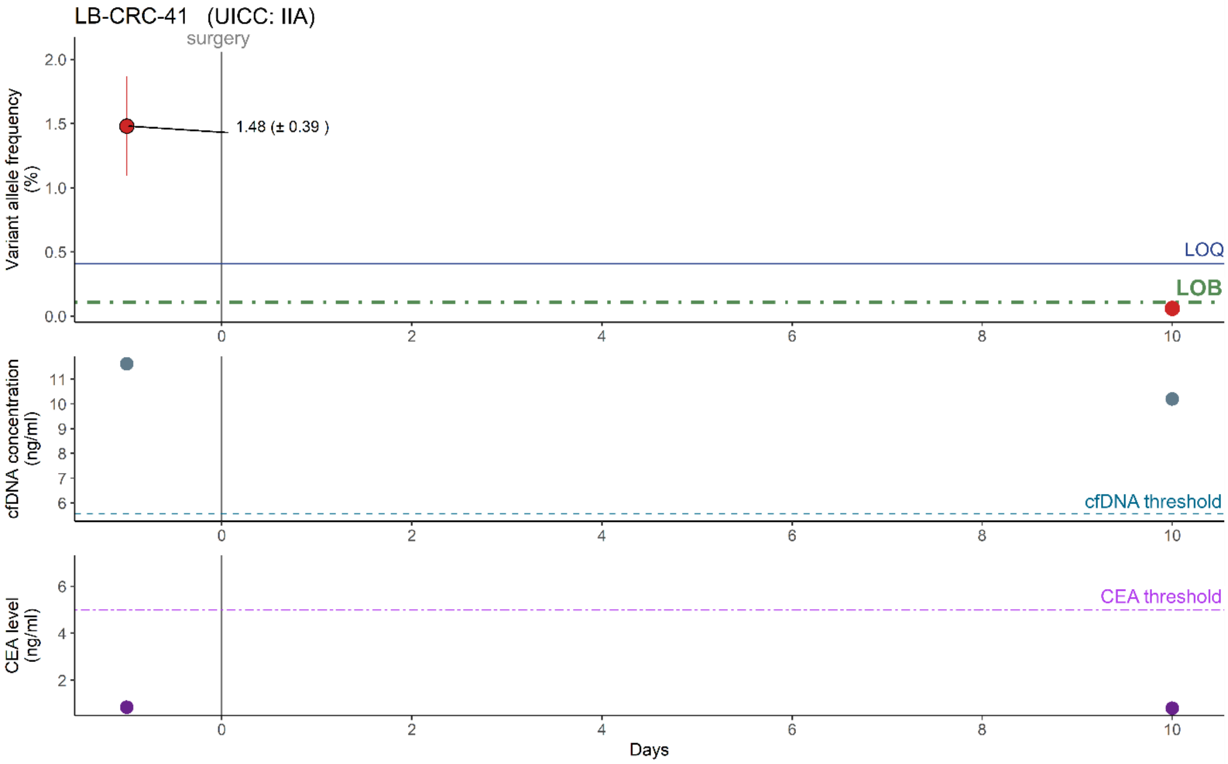

Supplementary Figure S18 Residual disease detection in patient LB-CRC-41.

Patient LB-CRC-41 was diagnosed with stage IIA CRC. ctDNA was present at baseline with a mutant VAF of 1.48% (>LOQ) and absent 10 days after surgery (<LOB).

Disease monitoring

LB-CRC-02

Patient LB-CRC-02 presented with a mutant VAF of 37.5% (±4.08%) at baseline. A good response to chemotherapy was predicted by a drop of mutant VAF <1%, which could be confirmed by the clinical diagnosis of partial remission approximately two and a half months after initiation of chemotherapy. This patient was diagnosed with progressive disease three months later and palliative chemotherapy was initiated. A correlation between mutant VAF and the diagnosis of progressive disease could not be established as no plasma samples were collected within this time period. Samples collected within the first two months after the start of palliative chemotherapy showed stable mutant VAF <1%. Approximately six months after initiation of palliative chemotherapy, mutant VAF increased to 18.61% (±2.03%), which may be related to newly developed resistance to chemotherapy. The patient died approximately six months after the first detection of increased mutant VAF. CEA levels and plasma cfDNA concentration were elevated in all plasma samples of this patient collected after initiation of palliative chemotherapy, which correlates well to the finding of progressive disease.

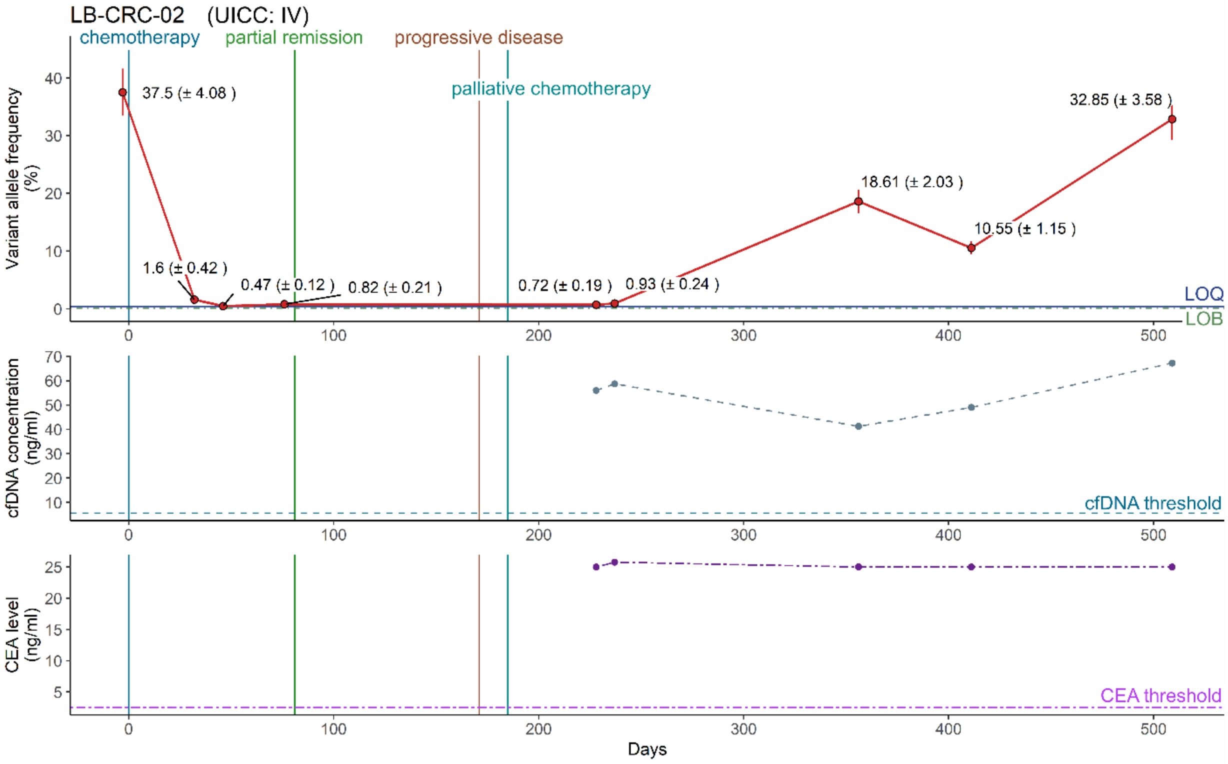

Supplementary Figure S19 *Monitoring of mutant variant, CEA levels and* cfDNA concentration in patient LB-CRC-02.

LB-CRC-07

No ctDNA was detected in patient LB-CRC-07 at baseline. About eight months after R0 resection a singular liver metastasis was identified, which was surgically removed. In parallel to the clinical finding of the distant metastasis, ctDNA was detected in plasma with a VAF of 0.53% (±0.19%). Following surgery, the mutant variant was not detectable anymore, but it reoccurred around 2.5 months after surgical removal of the liver metastasis. In a blood sample collected around two months later a mutant VAF of 0.82% (± 0.3) was detected. Another 2.5 months later systemic nodal progression was confirmed in the patient. Following this second disease recurrence, chemotherapy was initiated. 1.5 months after initiation of chemotherapy partial remission was clinically confirmed, which could be emphasized by a decreased mutant VAF below the LOQ, in a sample collected around 4.5 months after initiation of chemotherapy. Around one week prior to the diagnosis of stable disease the mutant variant was not detectable anymore in plasma. This result was confirmed by analysis of repeatedly collected samples throughout the course of chemotherapy. CEA levels obtained for all analyzed plasma samples, were in the normal range and provided therefore no evidence for disease recurrence. All plasma samples collected following the diagnosis of systemic nodal progression showed elevated cfDNA concentration, independently of clinical findings. In summary, ctDNA positive status was either a predictor of metastasis or recurrence at a later time point, which could not be translated to CEA and plasma cfDNA concentration. This highlights the potential of ctDNA to serve as predictive and prognostic biomarker.

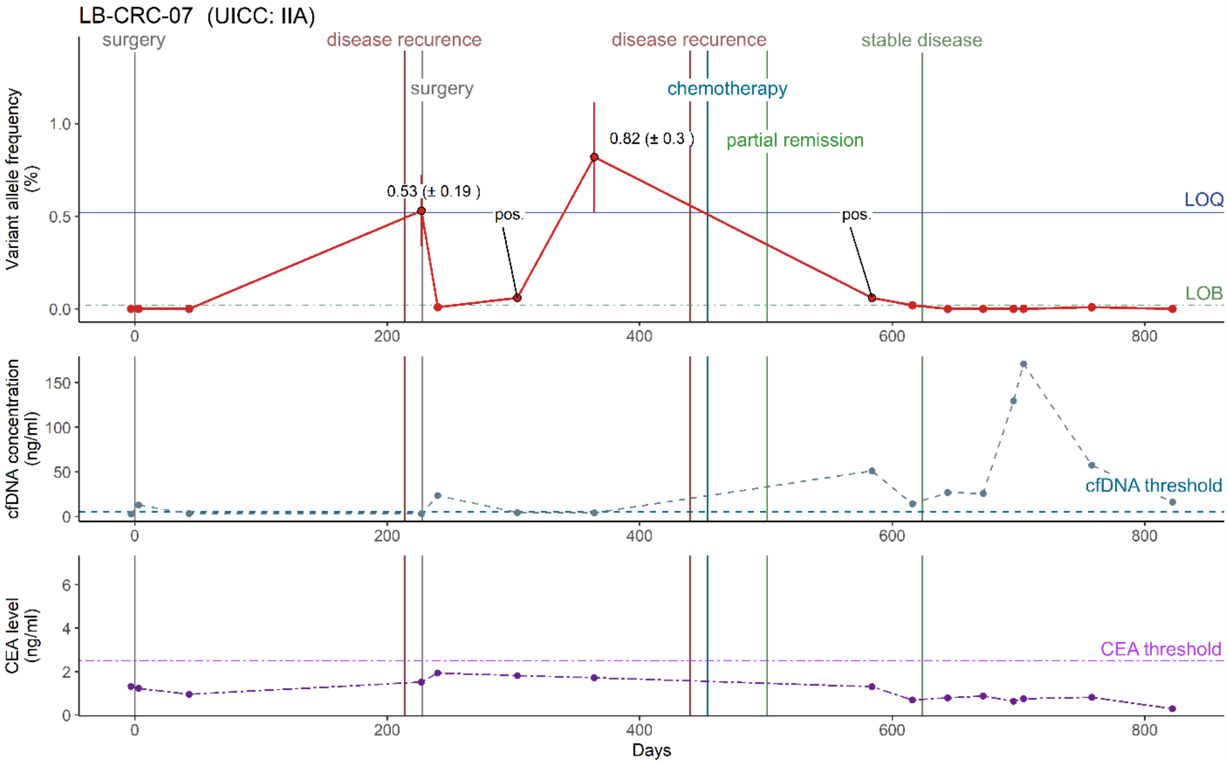

Supplementary Figure S20 Monitoring of mutant variant, CEA levels and cfDNA concentration in patient LB-CRC-07.

LB-CRC-18

No ctDNA was detectable at baseline in patient LB-CRC-18, diagnosed with stage III CRC. Further, the mutant variant could not be detected after surgery and did not occur during the course of adjuvant chemotherapy CEA levels were in the normal range and plasma cfDNA concentration was elevated in samples collected within 25 days after surgery.

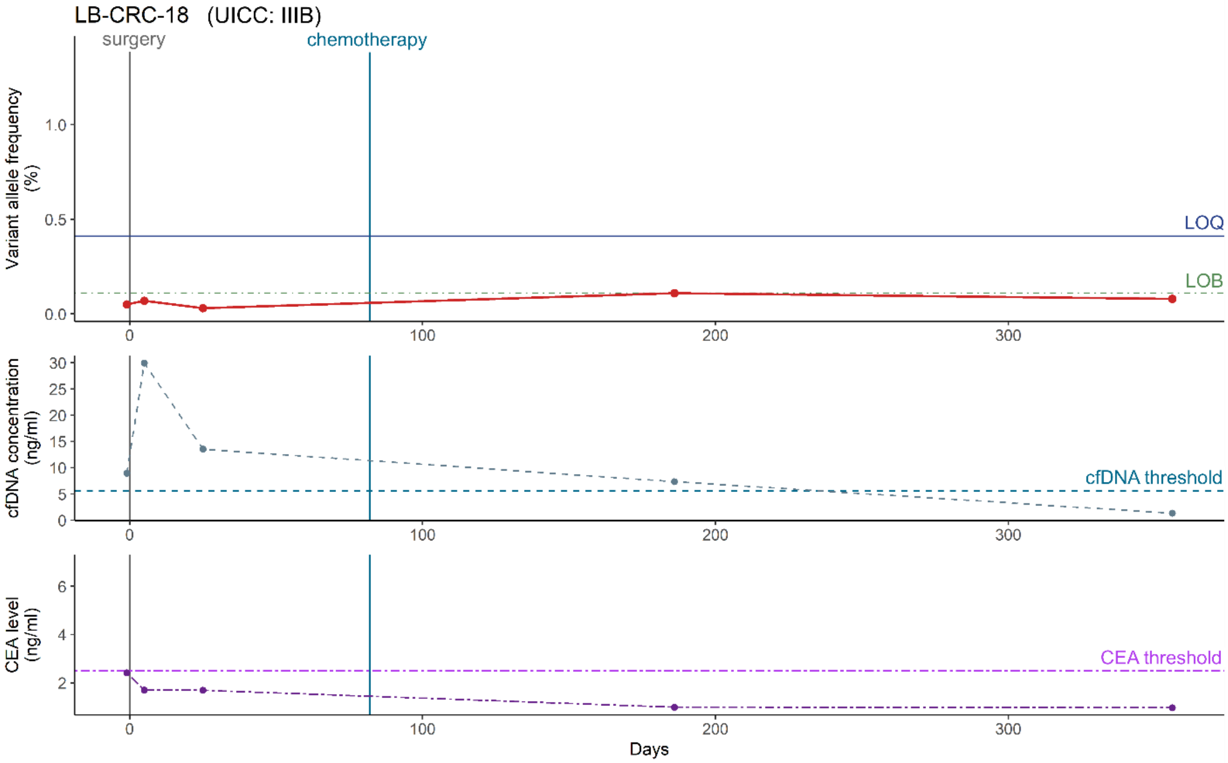

Supplementary Figure S21 Monitoring of mutant variant, CEA levels and cfDNA concentration in patient LB-CRC-18.

LB-CRC-25

In the baseline plasma sample of patient LB-CRC-25 ctDNA was detected. After primary surgery and locoregional R0 resection an increase of mutant VAF (0.52% (± 0.14%) and 1.72% (± 0.45%)) could be observed, without clinical evidence of residual disease or distant metastasis. Besides the detected mutant variant, CEA levels of these samples, were above the threshold of 2.5 ng/ml, being another indicator for potential residual disease. Around two months after surgery adjuvant chemotherapy was conducted over a time period of six months. Throughout the course of chemotherapy the mutant VAF decreased below the LOQ, and was not detectable around five months after completing chemotherapy. CEA levels of these samples were in the normal range. During follow-up, around six months after chemotherapy and one month after the last collected blood sample for this study, locoregional disease recurrence was observed. Unfortunately, no further blood samples could be obtained from the patient, to assess further changes in mutant VAF throughout the course of treatment. Plasma cfDNA concentration was in all but one sample elevated and therefore could not be correlated to clinical findings in this patient.

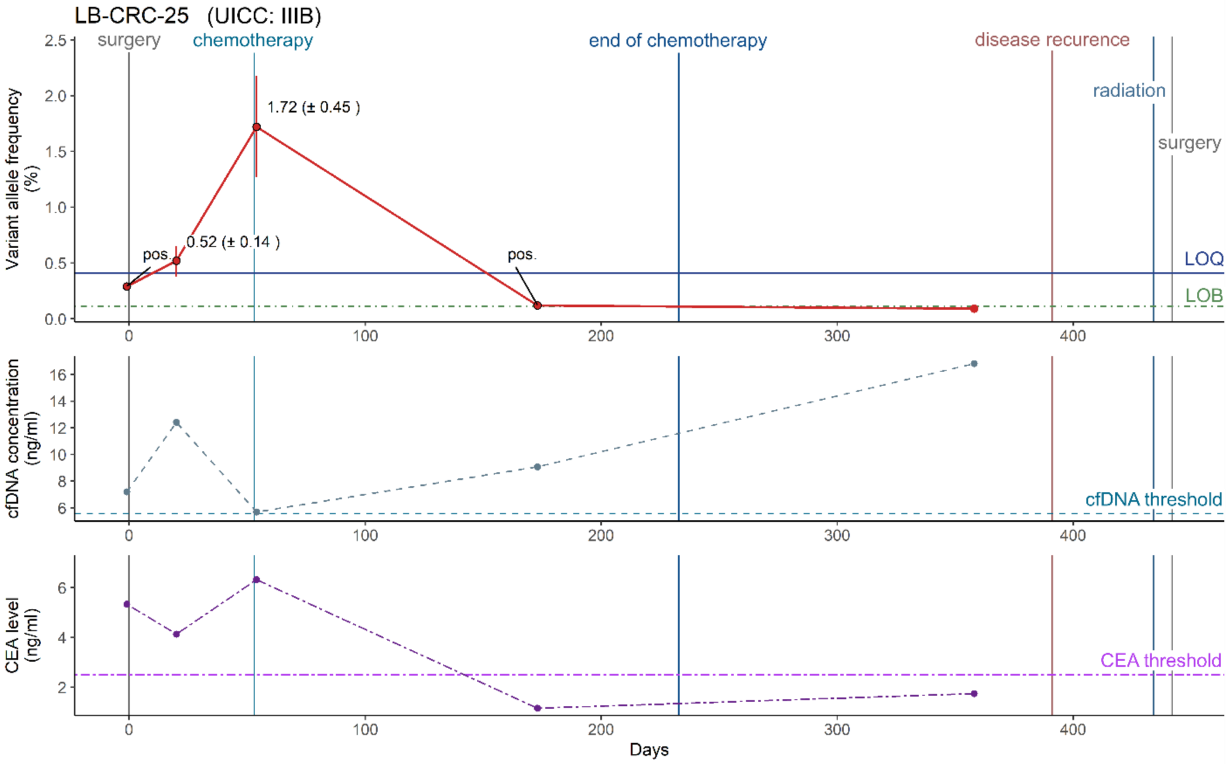

Supplementary Figure S22 Monitoring of mutant variant, CEA levels and cfDNA concentration in patient LB-CRC-25.

LB-CRC-29

In patient LB-CRC-29 ctDNA was detected at baseline. The mutant variant could not be detected anymore after surgery and did not recur during the course of adjuvant chemotherapy. CEA levels and plasma cfDNA concentration were in the normal range for all samples collected.

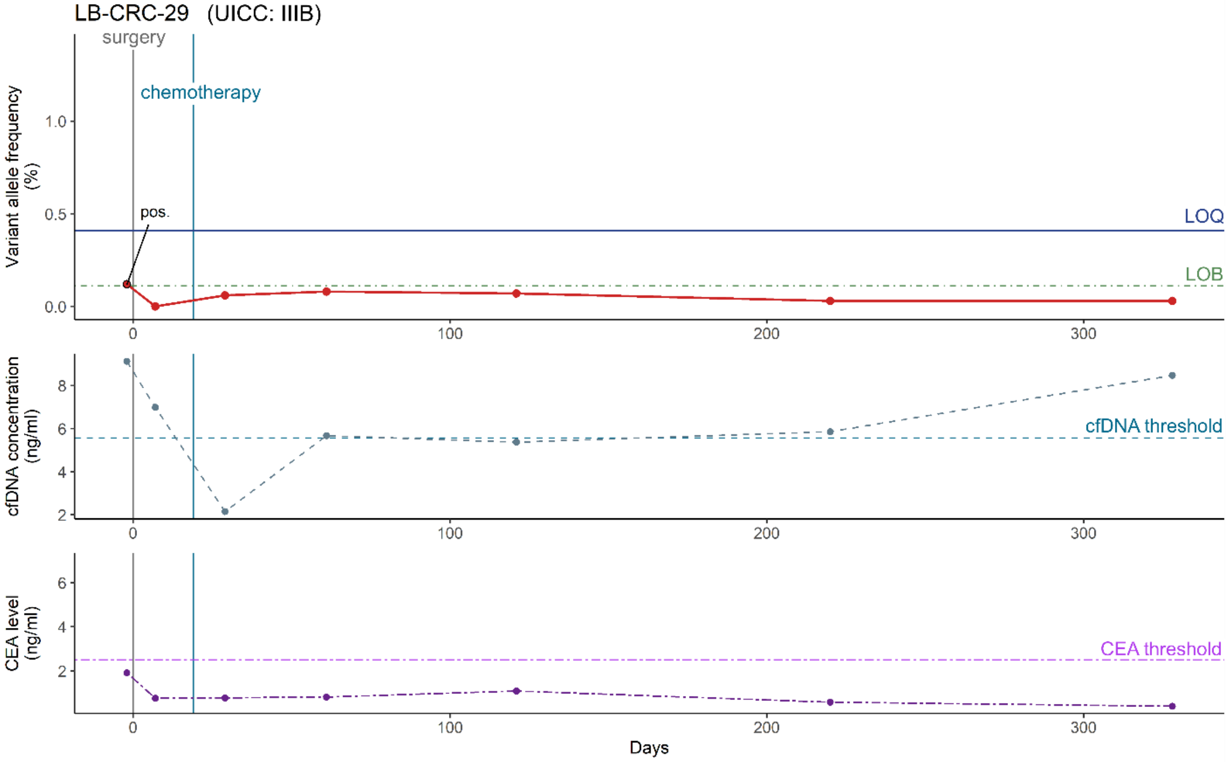

Supplementary Figure S23 Monitoring of mutant variant, CEA levels and cfDNA concentration in patient LB-CRC-29.

LB-CRC-30

Patient LB-CRC-30 presented with a mutant VAF of 19.33% (±2.88%) at baseline. After initiation of primary chemotherapy, mutant VAF decreased within the first three months (after three months below the LOQ), which correlates well with stable disease diagnosed after four and a half months. Only two weeks after this diagnosis, a significant increase in mutant VAF (13.28% (±1.98%)) was detected in the next blood sample, which dropped to <1%, within the next month and increased again to 11.06% (±1.65%) 1 month later. This increase in mutant VAF could be an indicator for resistance to chemotherapy, which was confirmed by the clinical diagnosis of progressive disease another month later. Elevated CEA levels were measured in all plasma samples collected from this patient. Plasma cfDNA concentration could not be correlated to the course of disease in this patient.

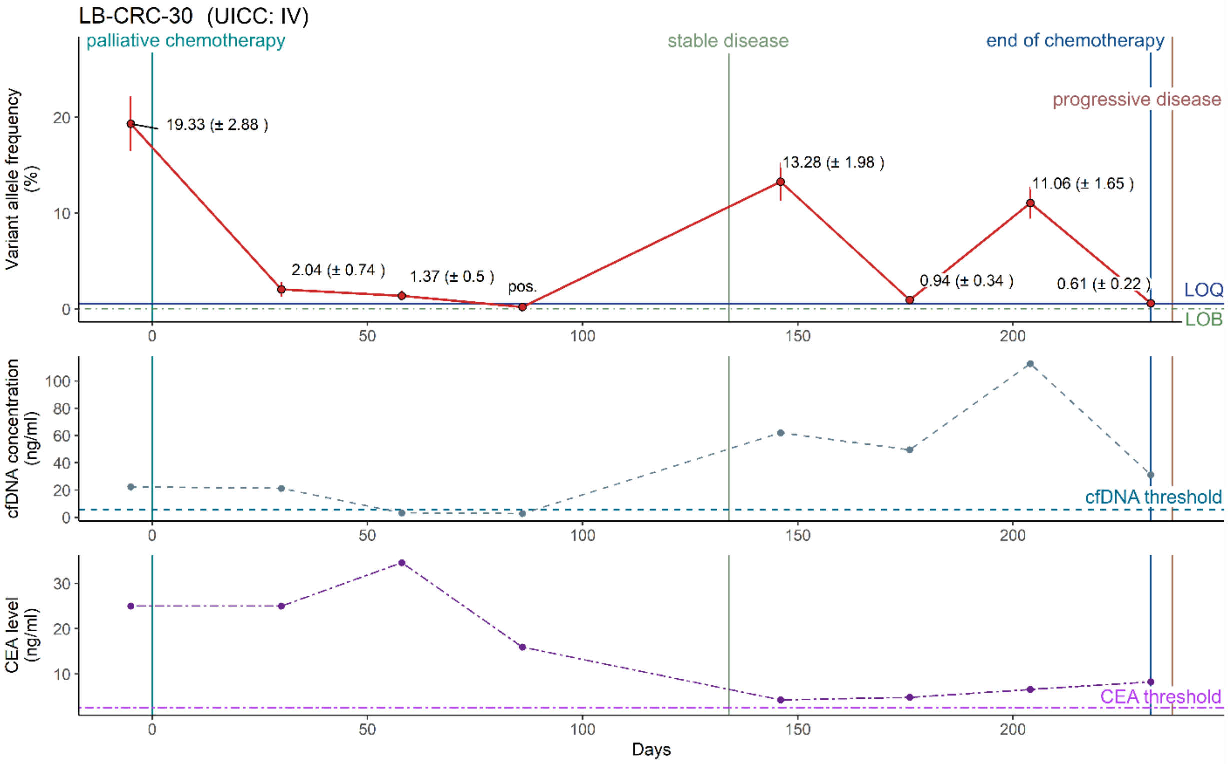

Supplementary Figure S24 Monitoring of mutant variant, CEA levels and cfDNA concentration in patient LB-CRC-30.

LB-CRC-32

Patient LB-CRC-32 presented with a mutant VAF of 47.75% (±7.11%) at baseline. The mutant variant was no longer detectable within the first two months after initiation of chemotherapy. No clinical findings were available that could be correlated to the results. CEA levels were at baseline, but decreased to normal levels approximately one month after the first absence of ctDNA. Over the subsequent four and a half months, the detectability of the mutant variant in plasma samples alternated between detectable and undetectable. Plasma cfDNA concentration was elevated in all plasma samples collected. Nevertheless, the absence of mutant variant and decrease in CEA levels could be an indicator for a good response to chemotherapy in this patient.

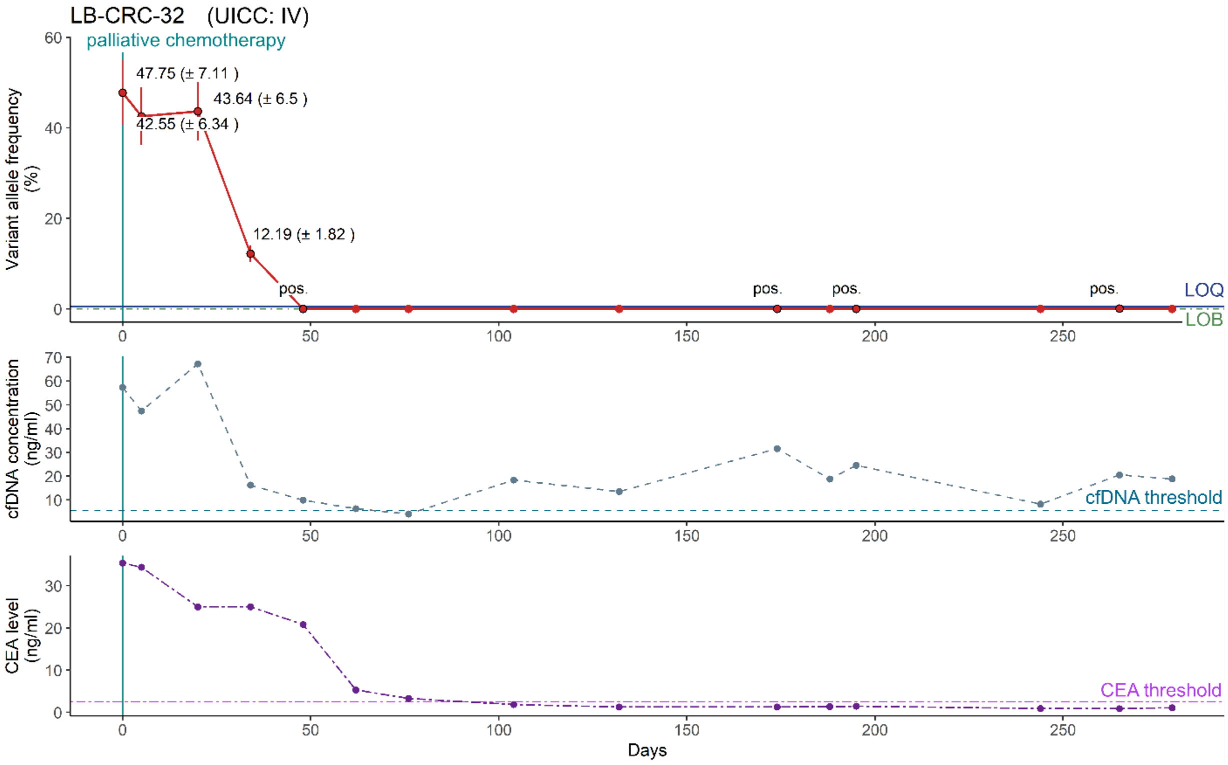

Supplementary Figure S25 Monitoring of mutant variant, CEA levels and cfDNA concentration in patient LB-CRC-32.

LB-CRC-38

A mutant VAF of 0.81% (±0.29%) was detected at baseline in patient LB-CRC-38. After surgery the mutant VAF increased to 2.01% (±0.73%) which could be correlated to the presence of metastasis. After initiation of chemotherapy, the mutant variant could no longer be detected within two months, which correlates well with the diagnosis of stable disease one month later. Interestingly, only in the first sample without ctDNA detection CEA levels were elevated, which could not be correlated to resistance to treatment. Similar to all patients described above, plasma cfDNA concentration was elevated in all samples collected.

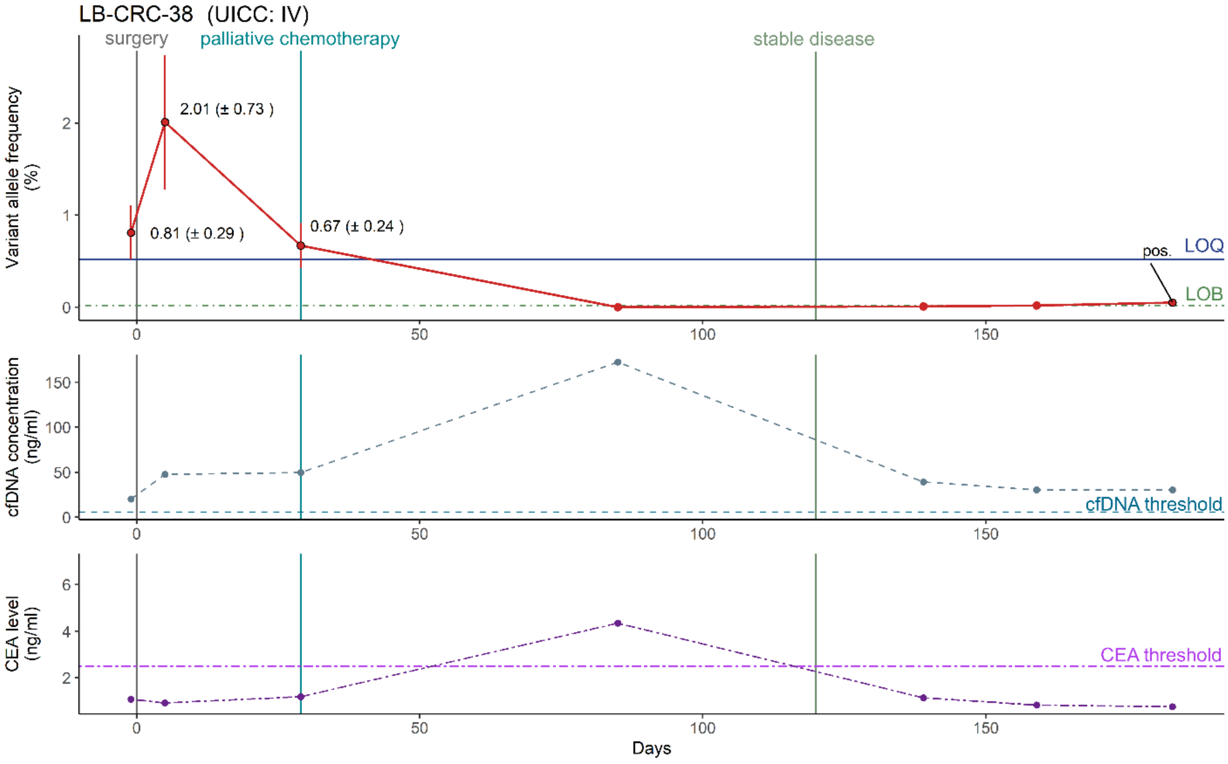

Supplementary Figure S26 Monitoring of mutant variant, CEA levels and cfDNA concentration in patient LB-CRC-38.

LB-CRC-43

Patient LB-CRC-43 presented with a mutant VAF of 8.99% (±2.36%) at baseline. The mutant variant was no longer detectable one month after initiation of primary chemotherapy. No clinical findings were available for this patient that could be correlated to the results. CEA levels were in the normal range and plasma cfDNA concentration was elevated in all plasma samples. The absence of mutant variant after initiation of chemotherapy could be an indicator for a good response to chemotherapy.

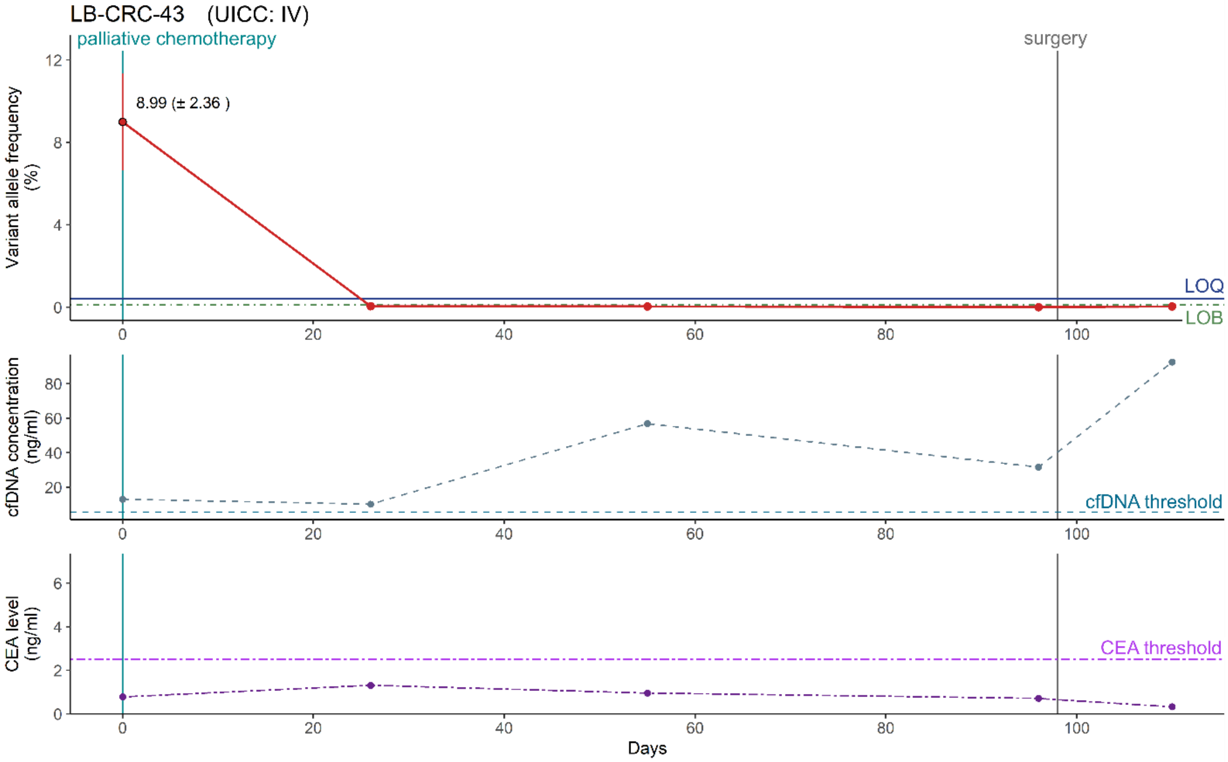

Supplementary Figure S27 Monitoring of mutant variant, CEA levels and cfDNA concentration in patient LB-CRC-43.

cfDNA concentration

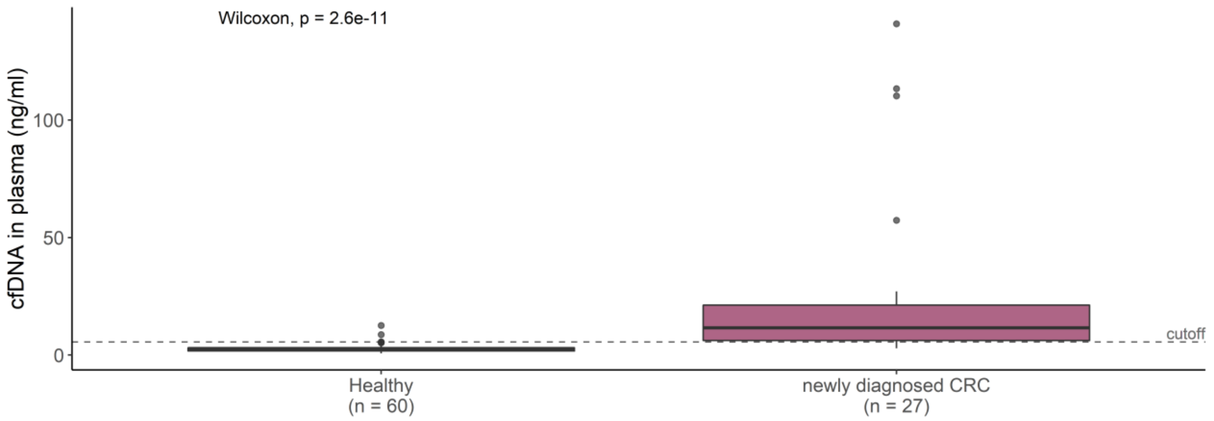

Supplementary Figure S28 Measurements of plasma cfDNA concentration in healthy individuals and newly diagnosed CRC patients for establishment of a CRC-specific cutoff for cfDNA elevation.
